# Warming and elevated CO_2_ promote rapid incorporation and degradation of plant‐derived organic matter in an ombrotrophic peatland

**DOI:** 10.1111/gcb.15955

**Published:** 2021-11-08

**Authors:** Nicholas O. E. Ofiti, Emily F. Solly, Paul J. Hanson, Avni Malhotra, Guido L. B. Wiesenberg, Michael W. I. Schmidt

**Affiliations:** ^1^ Department of Geography University of Zurich Zurich Switzerland; ^2^ Group for Sustainable Agroecosystems Department of Environmental Systems Science ETH Zurich Zurich Switzerland; ^3^ Environmental Sciences Division and Climate Change Science Institute Oak Ridge National Laboratory Oak Ridge Tennessee USA

**Keywords:** boreal peatland, decomposition, elevated CO_2_, lipid biomarker, organic matter, stable carbon isotope, warming

## Abstract

Rising temperatures have the potential to directly affect carbon cycling in peatlands by enhancing organic matter (OM) decomposition, contributing to the release of CO_2_ and CH_4_ to the atmosphere. In turn, increasing atmospheric CO_2_ concentration may stimulate photosynthesis, potentially increasing plant litter inputs belowground and transferring carbon from the atmosphere into terrestrial ecosystems. Key questions remain about the magnitude and rate of these interacting and opposing environmental change drivers. Here, we assess the incorporation and degradation of plant‐ and microbe‐derived OM in an ombrotrophic peatland after 4 years of whole‐ecosystem warming (+0, +2.25, +4.5, +6.75 and +9°C) and two years of elevated CO_2_ manipulation (500 ppm above ambient). We show that OM molecular composition was substantially altered in the aerobic acrotelm, highlighting the sensitivity of acrotelm carbon to rising temperatures and atmospheric CO_2_ concentration. While warming accelerated OM decomposition under ambient CO_2_, new carbon incorporation into peat increased in warming × elevated CO_2_ treatments for both plant‐ and microbe‐derived OM. Using the isotopic signature of the applied CO_2_ enrichment as a label for recently photosynthesized OM, our data demonstrate that new plant inputs have been rapidly incorporated into peat carbon. Our results suggest that under current hydrological conditions, rising temperatures and atmospheric CO_2_ levels will likely offset each other in boreal peatlands.

## Graphical abstract

Peatlands are a significant global soil carbon sinks and their response to warming and elevated CO_2_ is unclear. In an experimentally warmed and elevated CO_2_ boreal peatland, we investigated soil carbon responses in detail using novel techniques to understand soil carbon composition. We found that warming and drying altered peat soils in two main ways: (1) plant carbon inputs into peat increased and (2) decomposition rates increased. We demonstrate significant impact of global warming on naturally stored carbon and highlight its sensitivity to rising temperatures and atmospheric CO_2_ concentration.

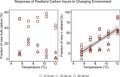

## 1 INTRODUCTION

More than one third of terrestrial soil carbon (C; 300–400 Pg C) is stored in boreal and subarctic peatlands, even though these ecosystems occupy less than 3% of the land surface (Bridgham et al., 2006; Gorham, 1991; Yu, 2012). Waterlogged conditions and the resultant lack of oxygen, combined with low temperatures and acidic pH hinder microbial decomposition resulting in net accumulation of organic matter (OM; Bragazza et al., 2013; Clymo, 1987; Freeman et al., 2001; Moore and Basiliko, 2006). Any environmental change that removes constraints on OM decomposition has the potential to alter the balance of these systems to either a smaller C accumulation rate or a net C loss (Gallego‐Sala et al., 2018).

The Intergovernmental Panel on Climate Change (IPCC) models project a 4–6°C increase in atmospheric and peat soil temperatures (by 2100, under RCP 8.5 scenario) in mid and high latitudes (IPCC, 2021; Soong et al., 2020). Rising temperatures are expected to enhance peatland heterotrophic respiration, potentially releasing substantial amounts of CO_2_ and CH_4_ to the atmosphere (Hopple et al., 2020; Wilson et al., 2016). Future climate warming is likely to be a consequence of rising atmospheric CO_2_ concentrations (Janzen, 2006). The projected climatic conditions are expected to stimulate photosynthesis, increase plant litter production, and transfer OM from the atmosphere into terrestrial ecosystems (Walker et al., 2020), potentially offsetting carbon losses through increased respiration (Keuper et al., 2017; Natali et al., 2011). However, there is substantial uncertainty about the interactive effects of warmer temperatures and rising CO_2_ concentrations on C cycling in peatlands (Ainsworth and Long, 2021; van Gestel et al., 2018). This is because the plant and microbial responses which regulate C cycling in peatlands (Buttler et al., 2015; Davidson and Janssens, 2006; Jassey et al., 2013; Pengerud et al., 2017; Sjögersten et al., 2016), may additionally be affected by climate‐induced changes in water‐table depth (Breeuwer et al., 2009; Laine et al., 2019; Pinsonneault et al., 2016). In particular, warming induced changes on water table depth, may have cascading effects on peat oxidation (decomposition; Freeman et al., 2001; Pinsonneault et al., 2016; Wilson et al., 2016). Warmer and drier conditions could stimulate C mineralization in surface aerobic layers of the acrotelm (Clymo and Bryant, 2008; Dorrepaal et al., 2009; Keuper et al., 2017), but these losses of C may be counterbalanced by increased aboveground plant productivity under elevated CO_2_ (Lu et al., 2013; Song et al., 2019; Yue et al., 2017). The anoxic catotelm (Clymo and Bryant, 2008) will likely not experience the drying that may occur in the acrotelm (Dorrepaal et al., 2009). Nevertheless, additional perturbations, such as lower water table (Zhong et al., 2020), increases in nutrient availability (primarily nitrogen) due to higher mineralization rates (Buttler et al., 2015), or changes in plant productivity (Dorrepaal et al., 2009; Keuper et al., 2017) could also drastically alter the C balance in the catotelm over time (Hopple et al., 2020; Wilson et al., 2016).

While changes in C stocks have been assessed in climate change experiments, these net changes do not provide information on mechanisms driving C dynamics (Bradford et al., 2016). Biomarker‐based proxies are powerful tools that can be used to understand these complex processes (Bailey et al., 2018). OM contains structurally unique molecular compounds that represent the decomposition products of plant and microbial biomass (Hall et al., 2020; Jansen & Wiesenberg, 2017; Kögel‐Knabner, 2017). Biomarkers can be used to follow qualitative and quantitative alterations in composition, sources and processes contributing to OM dynamics (Jansen & Wiesenberg, 2017). Global change experiments with CO_2_ enrichment provide an ideal setting for isotopic labelling of plant biomass, and examining OM transformation in intact ecosystems (Ainsworth & Long, 2021; Feng et al., 2010). Additionally, stable isotope signatures of molecular compounds offer opportunities for examining OM transformation of plant‐ and microbe‐derived biomass, representing molecules of different degradability and accessibility (Amelung et al., 2008; Feng et al., 2010).

Despite the established significance of peatlands in the global C cycle, we lack in situ evidence of how peat soils may respond to future climate change, since experiments that directly manipulate peat temperatures are rare and most studies only allow 1–2°C warming (Gill et al., 2017). To address this gap, the SPRUCE (Spruce and Peatland Responses Under Climatic and Environmental Change; https://mnspruce.ornl.gov/) experiment is assessing how boreal peatlands react to environmental change with an ecosystem‐scale climate manipulation (Hanson et al., 2017). The experiment adopts a multifactor regression design and incorporates aboveground and peat warming to five warming levels (+0, +2.25, +4.5, +6.75 and +9°C) repeated at both ambient and elevated CO_2_ concentrations (ambient and +500 ppm; Hanson et al., 2017). The combination of temperature and CO_2_ treatments at a range of increasing temperatures allows the study and evaluation of peat carbon cycling across mild to extreme scenarios for warming (Hanson et al., 2020; Hopple et al., 2020). The ^13^C depleted CO_2_ used in the elevated CO_2_ treatment, labels the exposed plant biomass with unique isotopic signatures and allowed us to trace its fate and examine OM transformations (Amelung et al., 2008).

In this study, we investigate the impact of warming (4 years of 0–9°C warming) and elevated CO_2_ (eCO_2_) concentration (2 years of +500 ppm CO_2_ addition) on OM composition and degradation at different depths at the SPRUCE experiment. Furthermore, we assessed the incorporation of ^13^C‐depleted carbon from plant‐derived inputs into OM under the eCO_2_ treatment. SPRUCE warming, to date, has resulted in lower water levels (10–30 cm below the hollow in the warmer plots) for longer durations during summer dry periods (Hanson et al., 2020) and increased CO_2_ and CH_4_ emissions (Gill et al., 2017; Hopple et al., 2020) and fine‐root growth (Malhotra et al., 2020). Overall, we hypothesized that increased fine‐root growth and lowered water table depth levels would stimulate decomposition and alter the quality of OM mainly in the acrotelm and less in the catotelm, especially at the warmest end of the SPRUCE temperature gradient. We further hypothesized that warming would accelerate peat decomposition through drying (Laine et al., 2019; Pinsonneault et al., 2016), and together with higher CO_2_ concentrations increase plant biomass inputs into OM (Malhotra et al., 2020; McPartland et al., 2019). To test these hypotheses, we employed molecular‐level analyses (biomarkers) that quantify specific plant‐ and microbe‐derived compounds (solvent extractable alkanes and fatty acids [FAs]). Furthermore, we analysed the isolated biomarkers for their compound‐specific stable carbon isotope signatures. This combination of analyses allowed us to trace the allocation of carbon belowground during the experimental period.

## 2 MATERIALS AND METHODS

### 2.1 Site description and experimental setup

The SPRUCE experimental site is located within the Marcell Forest in northern Minnesota, USA at the southern edge of the boreal zone (N 47°30.476′; W 93°27.162′; 412.7 to 413.1 m a.s.l.). The site has a subhumid continental climate with a mean annual temperature and precipitation of 3.4°C and 780 mm, respectively (between 1961 and 2010; Sebestyen et al., 2011). The bog is ombrotrophic (Typic Haplohemist soil), with peat depths of 2–3 m, and a pH ranging from 4.1 at the surface to 5.1 at 2 m (Sebestyen et al., 2011; Wilson et al., 2016). The overstory vegetation is composed of *Picea mariana* (black spruce) and *Larix laricina* (larch), whereas the understory is primarily composed of ericaceous shrubs (such as *Rhododendron groenlandicum* and *Chamaedaphne calyculata*), herbaceous species (*Maianthemum trifolium* and *Eriophorum vaginatum*) and *Sphagnum* mosses (Sebestyen et al., 2011; Tfaily et al., 2014; Wilson et al., 2016). The bog surface has a hummock and hollow microtopography (Nichols, 1998). To date, the water table has fluctuated about 30 cm relative to hollow during the growing seasons for this ongoing study (Hobbie et al., 2016; Iversen et al., 2018). The nominal boundary between the acrotelm (oxic layer) and mesotelm (layer where water table fluctuates) is at 30 cm depth, while for the boundary between the mesotelm and catotelm (anoxic layer below the water table) is at 30–75 cm depth, for more details see Hobbie et al. (2016) and references therein.

The SPRUCE experiment uses a regression‐based design that warms the vegetation and peat profile within ten octagonal transparent open‐top enclosures of 12 m diameter and a height of 7 m to five warming levels (*n* = 2 per treatment; +0, +2.25, +4.5, +6.75 and +9°C). The experiment warms peat down to a depth of ~3 m and was initiated between June and July 2014 by vertical installation of 3 m long heater cables placed inside plastic‐coated iron pipes. Heat exchangers, blowers and conduits were established in August 2015 to increase air temperatures, thereby warming the vegetation inside the enclosures (Hanson et al., 2017; Wilson et al., 2016). Temperature differentials in the enclosures are achieved by comparison to a single constructed‐control plot, and they are maintained during seasonal shifts throughout the year (Gill et al., 2017; Hanson et al., 2017). In June 2016, CO_2_ manipulation treatments were started, with duplicate warming plots receiving either ambient or elevated CO_2_ concentrations. The elevated CO_2_ treatment consists of elevating the local ambient CO_2_ concentration by +500 ppm (~900 ppm, with δ^13^C–CO_2_ isotope value of ~54‰; Hanson et al., 2017).

### 2.2 Peat and plant sampling and characterization

Peat cores (2 per plot) were collected in hollow microtopography from each of the plots in August 2018, where the surface of the hollow was defined as 0 cm. Surface samples (0–30 cm) were cut and extracted by hand, while deeper peat samples (30–200 cm) were collected using a Russian peat corer. Once collected, duplicate cores from the same plot were sectioned, homogenized and combined into 10 cm increments over 0 to 50 cm depth and 25 cm intervals from 50 to 200 cm. The peat samples were placed into plastic bags and stored frozen at −20°C immediately after sampling. Peat cores from +0, +2.25, +4.5, +6.75 and +9°C for the two CO_2_ treatments (ambient and elevated CO_2_) are presented in this manuscript.

Leaf tissues of the overstory‐ (*Picea* and *Larix*) and understory vegetation (shrubs and *Sphagnum*) were randomly collected from each plot (*n* = 2 per plant species). However, root tissues were collected only from the control plot to avoid disturbance within the treatment enclosures. Therefore, leaf tissue results from ambient temperature (+0°C) for the two CO_2_ treatments are presented here. All plant samples were kept frozen at −20°C until further analyses.

Peat sections and plant materials were freeze‐dried to constant weight. Peat samples were then passed through a 5‐mm sieve to remove larger litter fragments as 2‐mm sieve would have caused a loss of peat moss biasing the results. A subsample of the sieved peat and plant material was ground to a fine powder using a ball mill (MM400, Retsch). Organic carbon and nitrogen concentrations (%C, %N), as well as stable carbon and nitrogen isotope composition (δ^13^C, δ^15^N) were analysed by an Elemental Analyzer‐Isotope Ratio Mass Spectrometer (EA‐IRMS; Flash 2000‐HT Plus, linked by Conflo IV to Delta V Plus isotope ratio mass spectrometer, Thermo Fisher Scientific). Calibration was carried out using IAEA‐certified primary standards and caffeine (Merck), and a soil reference material originating from a Chernozem (Harsum) as secondary standard. Two analytical replicates were measured for all samples.

### 2.3 Solvent extractable lipid biomarker analysis

To characterize changes in OM quality, quantity and degradation, solvent extractable compounds were extracted from ~0.5–2 g of milled peat and plant material following the protocol by Wiesenberg and Gocke (2017). Total lipids were extracted using Soxhlet extraction with dichloromethane: methanol (93:7; v/v). The lipid extracts were separated into a neutral‐ and FA fraction (by eluting with dichloromethane and dichloromethane: formic acid (99:1; v/v), respectively) by solid phase separation using Silica 60 + 5% KOH, 63–200 μm (Macherey‐Nagel). Prior to gas chromatographic (GC) analysis, an aliquot of the FA fraction was spiked with deuterated eicosanoic acid (D_39_C_20_) as an internal standard and derivatized to fatty acid methyl esters (FAMEs) using boron trifluoride:methanol solution (BF_3_:MeOH). The alkanes were separated from the neutral fraction by elution with hexane using column chromatography (activated silica gel; 70–230 mesh, 100 Å) and spiked with deuterated tetracosane (D_50_C_24_) as an internal standard prior to GC analysis.

The alkanes and FAMEs were quantified on a GC (Agilent 7890B) equipped with a multimode injector and a flame ionization detector (FID). Analytical errors were typically <10% based on replicate analysis. Compound identification was performed on an Agilent 6890N GC equipped with split‐splitless injector coupled to an Agilent 5973 mass selective detector (MS). Individual compounds were identified by comparison of mass spectra with those of external standards and from the NIST and Wiley mass spectra library. Details of the GC operating conditions are described elsewhere (Ofiti et al., 2021). The data acquired were processed with Chemstation software. The concentrations of the target compounds were normalized to organic carbon concentration of the respective sample (stated as mg g^−1^ OC).

### 2.4 Compound‐specific isotope analysis

To examine OM incorporation and transformation in peat, compound‐specific δ^13^C analysis of individual *n*‐alkanes and FAMEs was performed using a Trace GC Ultra, coupled via GC Isolink II and Conflo IV to Delta V Plus isotope mass spectrometer (Thermo Fisher Scientific). The GC was equipped with a PTV injector and FID detector and DB‐5MS column (50 m × 0.2 mm × 0.33 μm) and 1.5 m pre‐column, with helium as the carrier gas (1 ml min^−1^). The sample (2 μl) was injected in splitless mode. The temperature program of the PTV injector increased from 80°C (held for 0.5 min) to 375°C (held for 2.5 min) at 870°C min^−1^ and reduced to 250°C at 50°C min^−1^. The GC oven temperature for *n*‐alkanes was held at 70°C for 4 min, and increased to 320°C at a rate of 5°C min^−1^, and held for 35 min. For FAMEs, the temperature was held at 50°C for 4 min, then increased to 150°C at a rate of 4°C min^−1^ and finally increased to 320°C at a rate of 3°C min^−1^ held for 25 min. The data acquired were processed with Isodat software. Reproducibility and stability (<0.5‰) of δ^13^C values were evaluated with pulses of CO_2_ reference gas and *n*‐alkane standard mixture (C_20, 24, 30, 32_; Sigma Aldrich) of known isotope composition, which was analysed in between every five samples. The δ^13^C abundance is expressed relative to the Vienna‐Pee Dee Belemnite (V‐PDB) reference standard in per mil (‰). Three analytical replicates were measured for all plant and peat (0–75 cm depths) samples. The difference between analytical replicates did not exceed 1.0‰.

### 2.5 Calculations

Microbe‐derived OM is characterized by shorter chain length than plant‐derived OM, due to the absence of any long‐chain FAs (>C_19_) and *n*‐alkanes (>C_24_; Harwood & Russell, 1984). Therefore, the average chain length (ACL) of alkanes and FAs can be used as molecular proxy for the source and degradation of OM (Wiesenberg et al., 2010). ACL was calculated as:

$$\text{ACL}=\sum \left(C_{n}*n\right)/\sum C_{n},$$

where *n* is the number of carbons and C*_n_* is the relative abundance of the respective compound with *n* carbons.

Fresh plant‐derived OM is characterized by odd‐over‐even dominance for *n*‐alkanes and even‐over‐odd dominance for FAs (Eglinton et al., 1962). The carbon preference index (CPI), thus, indicates input of mainly fresh OM (high CPI >10) or to which degree it has been degraded (values close to 1; Cranwell, 1981). CPI was calculated as:

$$\begin{array}{c} {\text{CPI}}_{\text{FA}}=\\ \left[\left(\sum C_{12‐32}\text{even}/\sum C_{13‐29}\text{odd}\right)+\left(\sum C_{12‐32}\text{even}/\sum C_{15‐31}\text{odd}\right)\right]/2, \end{array}$$

$$\begin{array}{c} {\text{CPI}}_{\text{ALK}}=\\ \left[\left(\sum C_{23‐33}\text{odd}/\sum C_{22‐30}\text{even}\right)+\left(\sum C_{23‐33}\text{odd}/\sum C_{24‐32}\text{even}\right)\right]/2, \end{array}$$

for *n*‐alkanes (CPI_ALK_), and FAs (CPI_FA_), respectively.

The isotopic composition of individual FA was corrected for the δ^13^C value of the methyl group that was added during derivatization (FAMEs) as:

$$\delta_{\text{UD}}=\frac{n+1}{n}\times \delta_{D}‐\frac{1}{n}\times \delta_{M},$$

where *n* is the number of C atoms in the underivatized FA and *δ*_UD_ and *δ*_D_ are the C isotope ratios of the underivatized and the derivatized FA, respectively. *δ*_M_ is the C isotope ratios of the added methyl group (+42.3 ± 0.1‰). *δ*_M_ was determined by repeated measurement of both derivatized and underivatized C_10_ and C_12_ FAs (*n* = 6).

The δ^13^C values for the most abundant *n*‐alkanes (C_23–33_), short‐chain (C_14–18_) and long‐chain (C_20–32_) FAs were separately calculated as weighed means of the isotope values of the individual *n*‐alkanes and FAs as:

$$\bar{\delta }=\sum_{i=a}^{b}w_{i}\times \delta_{i},$$

where *a* and *b* represent the lower and upper limits of the respective carbon number range, *w_i_* the relative abundance of the individual *n*‐alkane or FAs and *δ_i_* the isotope value of the individual *n*‐alkane or *n*‐FAs (Wiesenberg et al., 2008).

The fraction of new peat carbon that was derived from plant biomass input during the experimental period was calculated using a simple end‐member mixing model (Balesdent et al., 1988) as:

$$F_{\text{experiment ‐ derived}}=\frac{\delta_{\text{peat},\;\text{elevated}}‐\delta_{\text{peat},\;\text{ambient}}}{\delta_{\text{plant},\;\text{elevated}}‐\delta_{\text{plant},\;\text{ambient}}}\times 100,$$

where *δ*_peat, elevated_ and *δ*_peat, ambient_ are the δ^13^C values of organic carbon, *n*‐alkane or FAs for treatments with ^13^C‐depleted CO_2_ and ambient CO_2_, respectively. Corresponding, *δ*_plant, elevated_ and *δ*_plant, ambient_ are the δ^13^C values of organic carbon, *n*‐alkane or FAs in all aboveground plant biomass in the +0°C enclosures for treatments with ^13^C‐depleted CO_2_ and ambient CO_2_, respectively. For *δ*_plant_, mean isotope values of all aboveground plant biomass from each individual enclosure were used.

### 2.6 Statistical analyses

We evaluated the effects of temperature on peat organic carbon and nitrogen concentration and lipid biomarker by stepwise multiple linear regression analysis with temperature, elevated CO_2_, and elevated CO_2_ × temperature as possible factors. We visually (using scatter plots) verified that all bivariate relationships were linear. In the model, enclosure was treated as a random effect, while Akaike information criterion was used as the model selection condition to assess the ability of temperature and CO_2_ to predict lipid biomarkers. For instances in which both temperature and CO_2_ effects were significant, we evaluated separate regressions against temperature for ambient and elevated CO_2_ treatments. In the regression analysis we used the actual temperature measured at −0.3 m below the hollows averaged over the period 2016 to 2018 (Table S1). Normality and homoscedasticity in all models were visually checked using residuals and qqplots and adjusted when needed to fit parametric assumptions using log transformation. The chosen level of significance was 5% (*p* < .05) in all statistical tests.

Principal component analysis (PCA) was performed using the function *prcomp* in the R *stats* package to investigate patterns in the OM composition. The PCA data set included 110 data points with five variables. All variables were standardized before PCA analysis. All data analysis were performed using the R v.3.6.3 (R Core Team, 2020) using the RStudio interface v. 1.2.5033 (RStudio Team, 2019).

## 3 RESULTS

### 3.1 Organic carbon and nitrogen concentrations

**FIGURE 1:**
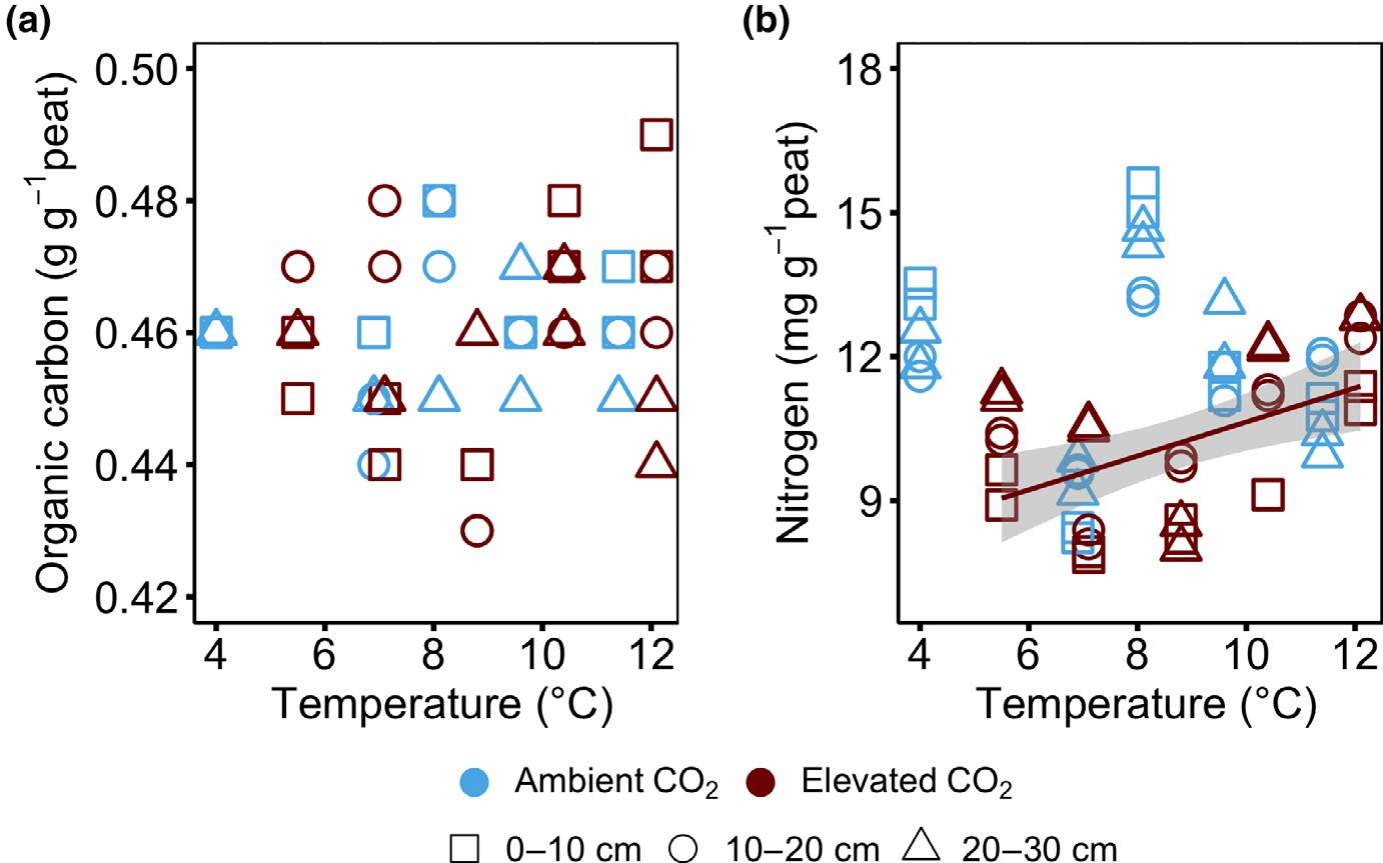
Peat organic matter responses to warming and elevated CO_2_ at 0–30 cm depth. (a) organic carbon, and (b) nitrogen concentrations following 4 years of warming and 2 years of elevated atmospheric CO_2_ concentrations. The concentration is plotted against average soil temperature measured at 0.3 m below the hollow surface from 2016 to 2018. Colours represent ambient (blue) or elevated CO_2_ (red) treatment. Symbols represent different sampling depth. Lines indicate significant treatment effects (regression smooth curves) *p* < .05. Linear regression with 95% confidence intervals is shown in grey. The absence of a line and/or confidence intervals indicates no significant trend. Under ambient CO_2_ treatment regression against temperature: (a) organic carbon, *r* ^2^ = .00, *p* = .88, *F* = 0.02, *df* = 28; (b) nitrogen, *r* ^2^ = .01, *p* = .62, *F* = 0.25, *df* = 28. Under elevated CO_2_ treatment regression against temperature: (a) organic carbon, *r* ^2^ = 0.01, *p* = .55, *F* = 0.36, *df* = 28; (b) nitrogen, *r* ^2^ = .23, *p* = .007, *F* = 8.42, *df* = 28

Peat organic carbon concentration increased with increasing depth, but there was no effect of temperature on the concentration in neither the ambient CO_2_ (aCO_2_) nor the elevated CO_2_ (eCO_2_) treatments when all depths or peat sequences were considered (i.e., acrotelm, 0–30 cm; mesotelm, 30–75 cm; and catotelm 75–200 cm; Figure 1a; Figure S1a).

Peat nitrogen concentrations in the +0°C enclosures were significantly lower in eCO_2_ than aCO_2_ treatments at 0–30 cm depth (*p* = .001; Figure 1b; Figure S1b). However, increasing temperature had no effects on the peat nitrogen concentrations under aCO_2_ treatments (*p* > .05; Figure S1b). By contrast, under eCO_2_ treatments, nitrogen concentrations increased linearly with increasing temperatures at 0–30 cm depth from 10.3 ± 0.4 mg g^−1^ in the +0°C enclosures to 12.2 ± 0.3 mg g^−1^ in the +9°C enclosures (*r* ^2^ = .23, *p* = .007; CO_2_ × temperature × depth interaction, *p* = .003; Figure 1b; Figure S1b). The observed changes in nitrogen concentrations were reflected in C:N ratios, which showed no response to warming under aCO_2_ treatment but increased linearly with increasing temperatures at 0–30 cm depth under eCO_2_ treatment (*r* ^2^ = .17, *p* = .02; Figure S1d).

The organic carbon and nitrogen concentrations of the plant biomass did not differ between aCO_2_ and eCO_2_ treatment in the +0°C enclosures (Table 1).

**TABLE 1:** Concentrations of organic carbon, nitrogen, *n*‐alkanes, branched fatty acids (FA), long‐chain and short‐chain *n*‐fatty acids (FA), and carbon preference index (CPI) of alkanes (CPI_ALK_) and fatty acids (CPI_FA_) and average chain length (ACL) of alkanes (ACL_ALK_) and fatty acids (ACL_FA_) and carbon isotope ratios (δ^13^C; ‰ V‐PDB) of aboveground (leaves) and belowground (roots) plant material, *n*‐alkanes and *n*‐fatty acids (FAs) from the trees (*Larix laricina* and *Picea mariana*), shrubs (*Rhododendron groenlandicum* and *Chamaedaphne calyculata*) and moss (*Sphagnum* spp.) from ambient temperature (+0°C) for the two CO_2_ treatments (ambient and elevated CO_2_; mean ± SE, *n* = 2)

	Ambient CO_2_				Elevated CO_2_			
	Aboveground plant biomass			Roots	Aboveground plant biomass			Roots
	Trees	Shrubs	Moss		Trees	Shrubs	Moss	
Organic carbon (mg g^−1^)	460.4 ± 6.7	479.0 ± 3.5	430.9 ± 4.9	447.0 ± 8.1	478.2 ± 3.7	491.0 ± 3.5	420.7 ± 3.8	nd
Nitrogen (mg g^−1^)	9.7 ± 1.5	11.9 ± 0.5	9.3 ± 0.1	3.8 ± 0.4	6.1 ± 0.3	10.4 ± 0.9	9.5 ± 0.2	nd
*n*‐Alkanes (mg g^−1^ OC)	0.4 ± 0.1	26.9 ± 11.1	0.9 ± 0.1*	0.4 ± 0.2	0.3 ± 0.1	24.8 ± 9.2	0.6 ± 0.1*	nd
Long‐chain *n*‐FA (mg g^−1^ OC)	10.4 ± 3.5	12.7 ± 5.7	10.2 ± 2.0	13.9 ± 2.3	11.7 ± 5.2	10.6 ± 4.1	8.5 ± 0.5	nd
Short‐chain *n*‐FA (mg g^−1^ OC)	16.8 ± 1.4	11.1 ± 0.6	10.4 ± 0.1*	6.8 ± 1.3	16.5 ± 1.5	9.2 ± 0.4	12.0 ± 0.6*	nd
Branched FA (mg g^−1^ OC)	bd	bd	bd	1.4 ± 1.2	bd	bd	bd	nd
ACL_ALK_	28.0 ± 0.1	31.0 ± 0.6	28.4 ± 0.4	29.8 ± 0.3	28.3 ± 0.5	31.0 ± 0.8	28.1 ± 0.2	nd
CPI_ALK_	6.8 ± 0.2	14.4 ± 3.1	13.0 ± 1.8	13.0 ± 0.4	9.0 ± 1.1	16.5 ± 0.2	13.3 ± 0.9	nd
ACL_FA_	19.6 ± 0.9	20.9 ± 1.1	20.2 ± 0.6	20.6 ± 0.9	19.7 ± 1.2	21.2 ± 0.9	19.5 ± 0.1	nd
CPI_FA_	16.6 ± 2.2	16.6 ± 2.1	10.0 ± 1.8	12.9 ± 1.9	17.0 ± 1.5	17.5 ± 1.8	8.2 ± 0.6	nd
δ^13^C of bulk OM (V‐PDB)	−29.2 ± 0.6	−28.8 ± 0.1	−29.8 ± 0.1	−28.4 ± 0.3	−44.6 ± 1.6	−46.0 ± 0.3	−43.7 ± 0.9	nd
δ^13^C of *n*‐alkanes (V‐PDB)	−30.7 ± 0.6	−32.8 ± 0.2	−38.1 ± 1.1*	−34.0 ± 0.2	−37.8 ± 0.2	−50.2 ± 0.2	−47.7 ± 0.4*	nd
δ^13^C of long‐chain *n*‐fatty acids (V‐PDB)	−33.5 ± 0.8	−37.3 ± 0.2	−41.4 ± 0.2*	−34.1 ± 1.5	−47.1 ± 1.1	−51.6 ± 0.1	−51.5 ± 0.2*	nd

Note

Significant treatment effects are indicated with asterisks: *p < .05.

Abbreviations: bd, below detection limit; nd, not determined.

### 3.2 Solvent‐extractable lipid biomarkers

**FIGURE 2:**
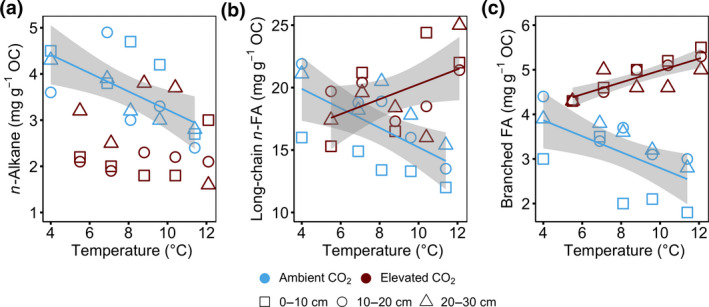
Relative abundance of plant‐ and microbial‐derived organic matter; (a) *n*‐Alkanes (C_23‐35_), (b) long‐chain *n*‐fatty acids (C_20–32_) and c) branched fatty acids (*iso*‐C_15_, *anteiso*‐C_15_, *iso*‐C_16_, *iso*‐C_17_, *anteiso*‐C_17_, *cis9,10*‐cy‐C_16_, *cis9,10*‐cy‐C_18_ and C_18:2ω6_) at 0–30 cm depth increment following 4 years of warming and 2 years of elevated atmospheric CO_2_ concentrations. The concentration is plotted against average soil temperature measured at 0.3 m below the hollow surface from 2016 to 2018. Colours represent ambient (blue) or elevated CO_2_ (red) treatment. Symbols represent different sampling depth. Lines indicate significant treatment effects *p* < .05. Linear regression with 95% confidence intervals is shown in grey. The absence of a line and/or confidence intervals indicates no significant trend. Under ambient CO_2_ treatment regression against temperature: (a) *n*‐Alkanes, *r* ^2^ = .44, *p* = .007, *F* = 10.04, *df* = 13; (b) Long‐chain *n*‐fatty acids, *r* ^2^ = .43, *p* = .008, *F* = 9.69, *df* = 13; (c) branched fatty acids, *r* ^2^ = .39, *p* = .01, *F* = 8.32, *df* = 13. Under elevated CO_2_ treatment regression against temperature: (a) *n*‐Alkanes, *r* ^2^ = .00, *p* = .94, *F* = 0.01, *df* = 13; (b) Long‐chain *n*‐fatty acids, *r* ^2^ = .25, *p* = .059, *F* = 4.27, *df* = 13; (c) branched fatty acids, *r* ^2^ = .69, *p* = .0001, *F* = 28.87, *df* = 13

Warming and elevated CO_2_ treatments altered the OM composition, although not all depths responded similarly. The *n*‐alkane (C_23–35_) concentrations (as a biomarker for plant‐derived OM) in the +0°C enclosures were significantly higher under aCO_2_ than eCO_2_ treatment at 0–30 cm depth (*p* = .02; Figure 2a). The *n*‐alkane concentrations under aCO_2_ treatment decreased linearly with increasing temperature at 0–30 cm depth (*r* ^2^ = .44, *p* = .007; Figure 2a). However, there was no effect of increasing temperature on the *n*‐alkane concentrations under the eCO_2_ treatment (*p* > .05; Figure 2a; Figure S2a).

Plant‐derived long chain *n*‐FA (C_20–32_) concentrations did not differ between aCO_2_ and eCO_2_ treatments in the +0°C enclosures (*p* > .05; Figure 2b; Figure S2b). However, the concentrations at 0–30 cm depth decreased linearly with increasing temperatures under aCO_2_ treatment (*r* ^2^ = .43, *p* = .008; Figure 2b) but increased linearly with increasing temperatures under eCO_2_ treatment (*r* ^2^ = .25, *p* = .059; Figure 2b). The observed changes in long chain *n*‐FA concentrations were echoed by plant‐ and microbe‐derived short chain *n*‐FA (C_12–18_) concentrations, which showed no response to warming under eCO_2_ treatment, but increased linearly with increasing temperatures at 0–30 cm depth under aCO_2_ treatment (*r* ^2^ = .28, *p* = .04; Figure S3a).

Branched FAs (*iso*‐C_15_, *anteiso*‐C_15_, *iso*‐C_16_, *iso*‐C_17_, *anteiso*‐C_17_, *iso*‐C_18_, *iso*‐C_19_, *cis9,10*‐cy‐C_16_, *cis9,10*‐cy‐C_18,_ C_18:2ω6_) concentrations (as a biomarker for microbe‐derived OM) at 0–30 cm depth was significantly higher in eCO_2_ than aCO_2_ treatments across all enclosures (*p* < .05; Figure 2c) and decreased linearly with increasing temperatures under aCO_2_ treatment (*r* ^2^ = .39, *p* = .01; temperature × depth interaction, *p* = .039; Figure 2c), but increased linearly with increasing temperatures under eCO_2_ treatment (*r* ^2^ = .69, *p* = .0001; Figure 2c).

Neither *n*‐alkane nor long‐chain *n*‐FA concentrations in the plant biomass differed between aCO_2_ and eCO_2_ treatment in the +0°C enclosures. However, under aCO_2_ treatment, *n*‐alkane and long‐chain *n*‐FA concentrations differed between plant components and were less abundant in roots than in leaves (Table 1). Branched FAs were only observed in plant roots under aCO_2_ treatment (Table 1).

**FIGURE 3:**
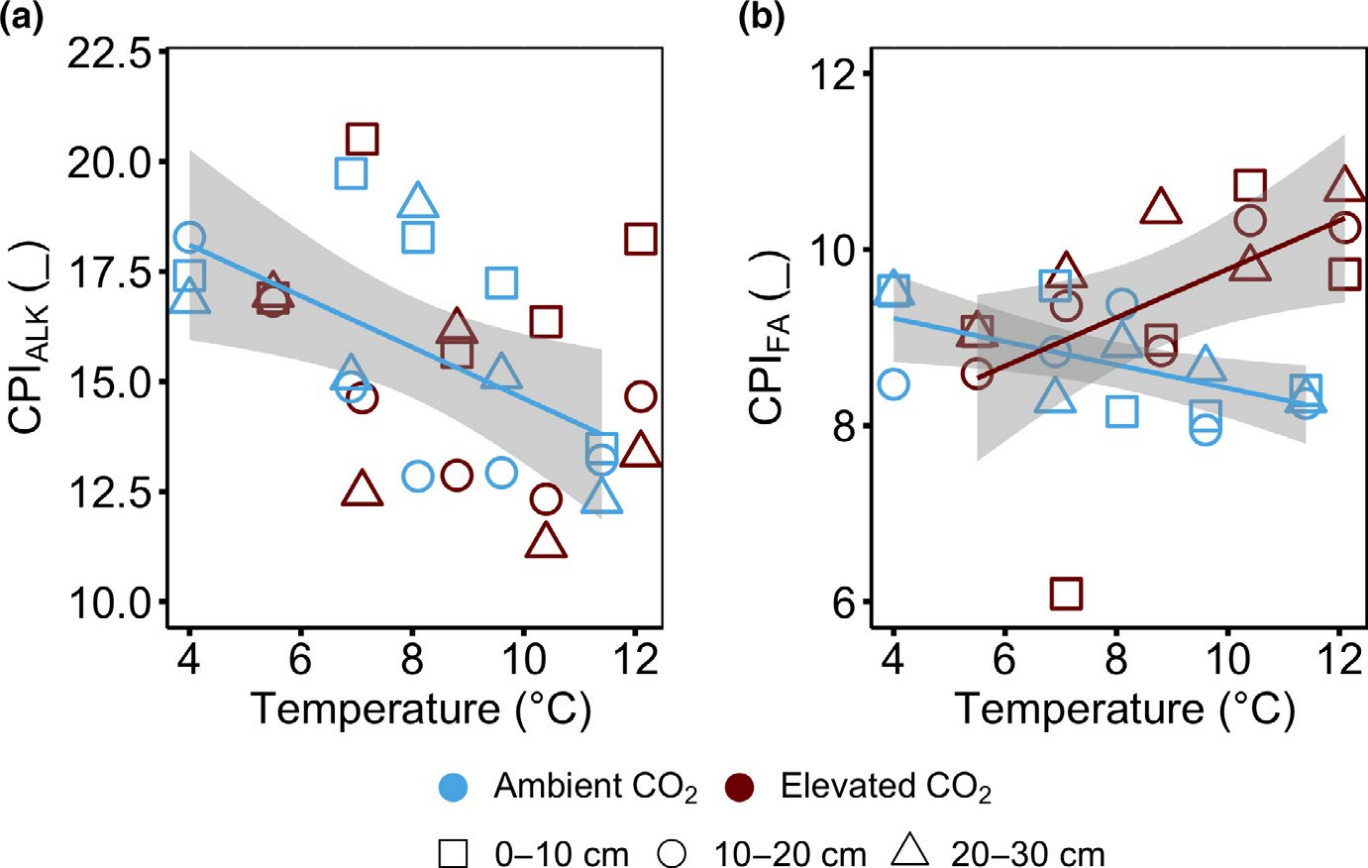
Organic matter decomposition proxy of carbon preference index (CPI) of a) *n*‐Alkane (CPI_ALK_) and b) fatty acids (CPI_FA_) at 0–30 cm depth following 4 years of warming and 2 years of elevated atmospheric CO_2_ concentrations. CPI is plotted against average soil temperature measured at 0.3 m below the hollow surface from 2016 to 2018. Positive decomposition responses from peat samples collected at 0–30 cm below the hollow surface. Colours represent ambient (blue) or elevated CO_2_ (red) treatment. Symbols represent different sampling depth. Lines indicate significant treatment effects (regression smooth curves) *p* < .05. Linear regression with 95% confidence intervals is shown in grey. The absence of a line and/or confidence intervals indicates no significant trend. Under ambient CO_2_ treatment regression against temperature: a) CPI_ALK_, *r* ^2^ = .37, *p* = .02, *F* = 7.53, *df* =13; b) CPI_FA_, *r* ^2^ = .36, *p* = .02, *F* = 7.39, *df* =13. Under elevated CO_2_ treatment regression against temperature: a) CPI_ALK_, *r* ^2^ = .10, *p* = .25, *F* = 1.46, *df* =13; b) CPI_FA_, *r* ^2^ = .33, *p* = .03, *F* = 6.40, *df* =13

We used the CPI as a decomposition proxy. Both *n*‐alkanes (CPI_ALK_) and FA (CPI_FA_) proxies did not differ between aCO_2_ and eCO_2_ treatments in the +0°C enclosures (*p* > .05; Figure 3a,b; Figure S4c,d). However, at 0–30 cm depth, CPI_ALK_ decreased linearly with increasing temperature under aCO_2_ treatments from 17.5 ± 0.4 in the +0°C enclosures to 13.0 ± 0.4 in the +9°C enclosures (*r* ^2^ = .37, *p* = .02; Figure 3a) but showed no response to warming under eCO_2_ treatment (Figure 3a; Figure S4c). Similarly, CPI_FA_ at 0–30 cm decreased linearly with increasing temperatures under aCO_2_ treatment (*r* ^2^ = .36, *p* = .02; Figure 3b) but increased linearly with increasing temperatures under eCO_2_ treatment (*r* ^2^ = .33, *p* = .03; CO_2_ × temperature × depth interaction, *p* = .077; Figure 3b). Overall, there was no effect of temperature on the ACL of *n*‐alkanes (ACL_ALK_) and FAs (ACL_FA_) in aCO_2_ and eCO_2_ treatments when considering all depths (Figure S4a,b). However, ACL_FA_ decreased linearly with increasing temperature at 0–30 cm depth under aCO_2_ treatment (*r* ^2^ = .37, *p* = .02; Figure S4b).

In the plant biomass, CPI (CPI_ALK_ and CPI_FA_) and ACL (ACL_ALK_ and ACL_FA_) did not differ between aCO_2_ and eCO_2_ treatment or between individual plant components in the +0°C enclosures (Table 1).

### 3.3 Carbon isotope composition

**FIGURE 4:**
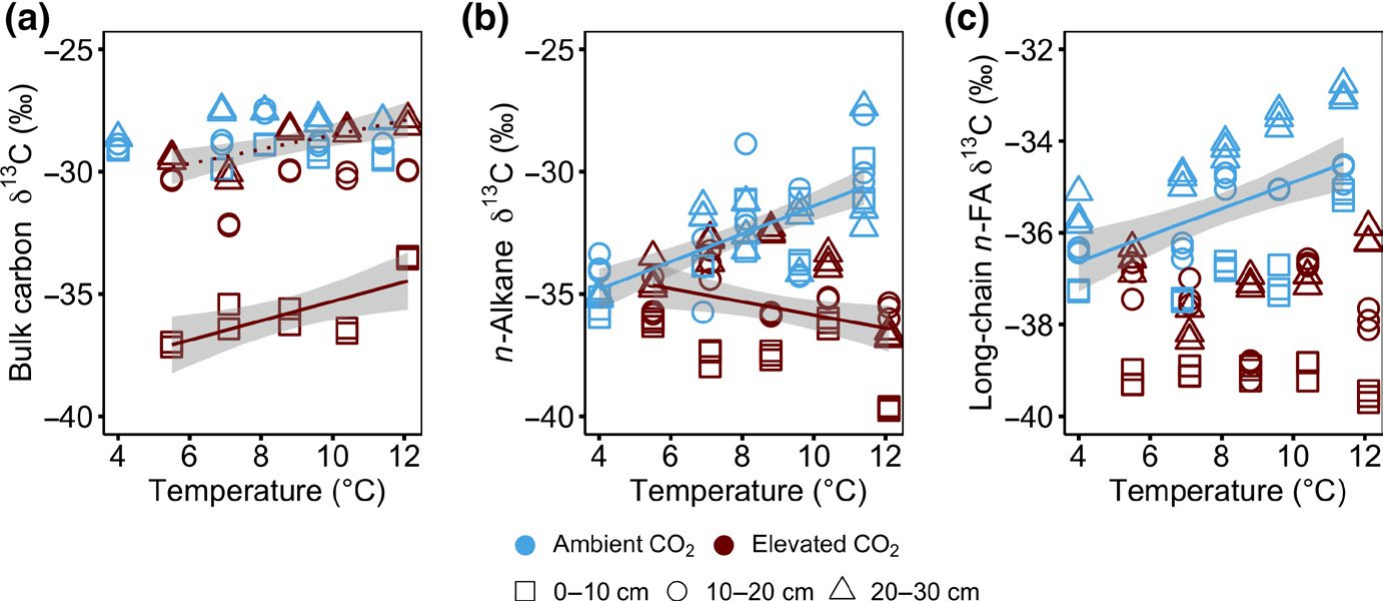
The δ^13^C values of (a) bulk organic matter (OM), and the most abundant (b) *n*‐Alkane (C_23–33_) and (c) long‐chain *n*‐fatty acids (C_20–32_) given in ‰ V‐PDB at 0–30 cm depth increment following 4 years of warming and 2 years of elevated atmospheric CO_2_ concentration. The isotopic difference between ambient and ^13^C‐depleted CO_2_ was c. 6‰ V‐PDB. The δ^13^C values are plotted against average soil temperature measured at 0.3 m below the hollow surface from 2016 to 2018. Colours represent ambient (blue) or elevated CO_2_ (red) treatment. Symbols represent different sampling depth. Lines indicate significant treatment effects *p* < .05. Linear regression with 95% confidence intervals is shown in grey. The absence of a line and/or confidence intervals indicates no significant trend. Under ambient CO_2_ treatment regression against temperature: (a) bulk OM, *r* ^2^ = .01, *p* = .66, *F* = 0.20, *df* = 28; (b) *n*‐Alkane, *r* ^2^ = .49, *p* < .0001, *F* = 40.83, *df* = 43; (c) long‐chain *n*‐fatty acids, *r* ^2^ = .29, *p* = .0001, *F* = 17.71, *df* = 43. Under elevated CO_2_ treatment regression against temperature: (a) bulk OM at 0–10 cm (full line), *r* ^2^ = .55, *p* = .01, *F* = 9.97, *df* = 8 and at 20–30 cm (dotted line), *r* ^2^ = .65, *p* = .005, *F* = 14.81, *df* = 8; (b) *n*‐Alkane, *r* ^2^ = .11, *p* = .02, *F* = 5.43, *df* = 43; (c) long‐chain *n*‐fatty acids, *r* ^2^ = .00, *p* = .94, *F* = 0.01, *df* = 43

Overall, the δ^13^C values of bulk OM, *n*‐alkanes (C_23–33_) and long‐chain *n*‐FAs (C_20–30_) were more depleted at 0–30 cm depth in the eCO_2_ than the aCO_2_ treatment (1.7‰–5.0‰ for bulk OM, 0.5‰–7.2‰ for *n*‐alkanes and 1.2‰–3.6‰ for *n*‐FAs; *p* < .05; Figure 4). Furthermore, total lipid extracts were c. 5–8‰ more depleted in δ^13^C values compared with bulk OM (Figure 4). The δ^13^C values of bulk OM did not differ throughout the peat sequences under aCO_2_ treatment (Figure 4a) but increased linearly with increasing temperatures in *n*‐alkane and long‐chain *n*‐FA fractions at 0–30 cm depth (*r* ^2^ = .49, *p* < 0.0001; temperature × depth interaction, *p* = .09; and *r* ^2^ = .29, *p* = .0001; temperature × depth interaction, *p* = .004 in *n*‐alkane and *n*‐FA fractions, respectively; Figure 4b,c). Under eCO_2_ treatments, ^13^C values of bulk OM increased linearly with increasing temperatures at 0–10 cm depth (*r* ^2^ = .55, *p* = .01) and 20–30 cm depth (*r* ^2^ = .65, *p* = .005; CO_2_ × temperature × depth interaction, *p* = .0002; Figure 4a), but decreased linearly with increasing temperatures in *n*‐alkane at 0–30 cm depth (*r* ^2^ = .11, *p* = .025; CO_2_ × temperature × depth interaction, *p* = .045; Figure 4b). However, ^13^C abundances of long‐chain *n*‐FAs did not differ throughout the peat sequences under eCO_2_ treatment (Figure 4c; Figure S5c).

The δ^13^C values of bulk carbon, *n*‐alkanes and *n*‐FAs were c. 13 ‰ higher in plant biomass under aCO_2_ than eCO_2_ treatment in the +0°C enclosures (*p* < .05; Table 1), but were similar between individual plant components and were within the typical range for C3 plant tissues.

### 3.4 Estimation of the incorporation of labelled carbon into organic matter

**FIGURE 5:**
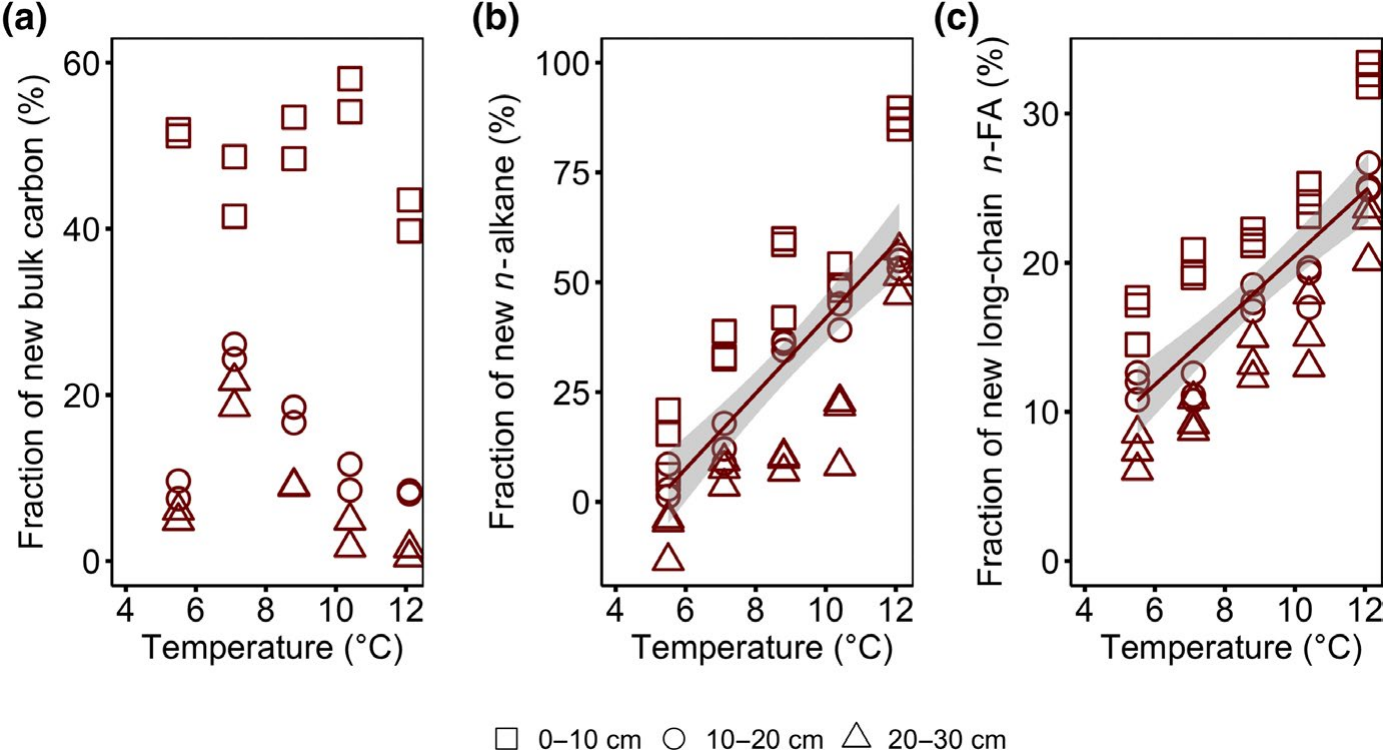
The contribution of experimentally derived plant carbon to (a) bulk organic matter (OM), (b) *n*‐Alkane (C_23–33_) and (c) long‐chain *n*‐fatty acids (C_20–32_) at 0–30 cm depth increment after 4 years of warming and 2 years of elevated atmospheric CO_2_ concentrations. The proportion of new (experiment‐derived) carbon is plotted against average soil temperature measured at 0.3 m below the hollow surface from 2016 to 2018. Symbols represent different sampling depth. Lines indicate significant treatment effects *p* < .05. Linear regression with 95% confidence intervals is shown in grey. The absence of a line and/or confidence intervals indicates no significant trend. Regression against temperature: (a) bulk OM, *r* ^2^ = .02, *p* = .51, *F* = 0.44, *df* = 28; (b) *n*‐Alkane, *r* ^2^ = .63, *p* < .0001, *F* = 72.38, *df* = 43; (c) long‐chain *n*‐fatty acid, *r* ^2^ = .58, *p* < .0001, *F* = 60.36, *df* = 43

Differences between δ^13^C values of ambient and ^13^C‐depleted eCO_2_ treatments in bulk OM, *n*‐alkanes and long‐chain *n*‐FAs increased with increasing temperature (from 1.7, 0.4 and 1.2‰ in +0°C plot to 5.0, 7.2, and 3.6‰ in the +9°C plot for bulk OM, *n*‐alkanes and *n*‐FAs, respectively). The proportions of experiment‐derived carbon increased in the order total organic carbon < long‐chain FAs < *n*‐alkanes < short‐chain FAs (Figure 5; Figure S6). Although bulk OM did not show significant effects of temperature on the proportions of experiment‐derived carbon, the *n*‐alkane and long‐chain FA did. For both, the proportions of experiment‐derived carbon increased linearly with temperature at 0–30 cm depth (from 3.7 ± 6.2% in the +0°C plot to 64.3 ± 11.3% in the +9°C plot; *r* ^2^ = .63, *p* < .0001 for *n*‐alkanes and from 11.8 ± 2.6% in the +0°C plot to 26.8 ± 3.1% in the +9°C plot; *r* ^2^ = .58, *p* < .0001 for long‐chain FAs; Figure 5b,c).

### 3.5 Changes in organic matter molecular composition

**FIGURE 6:**
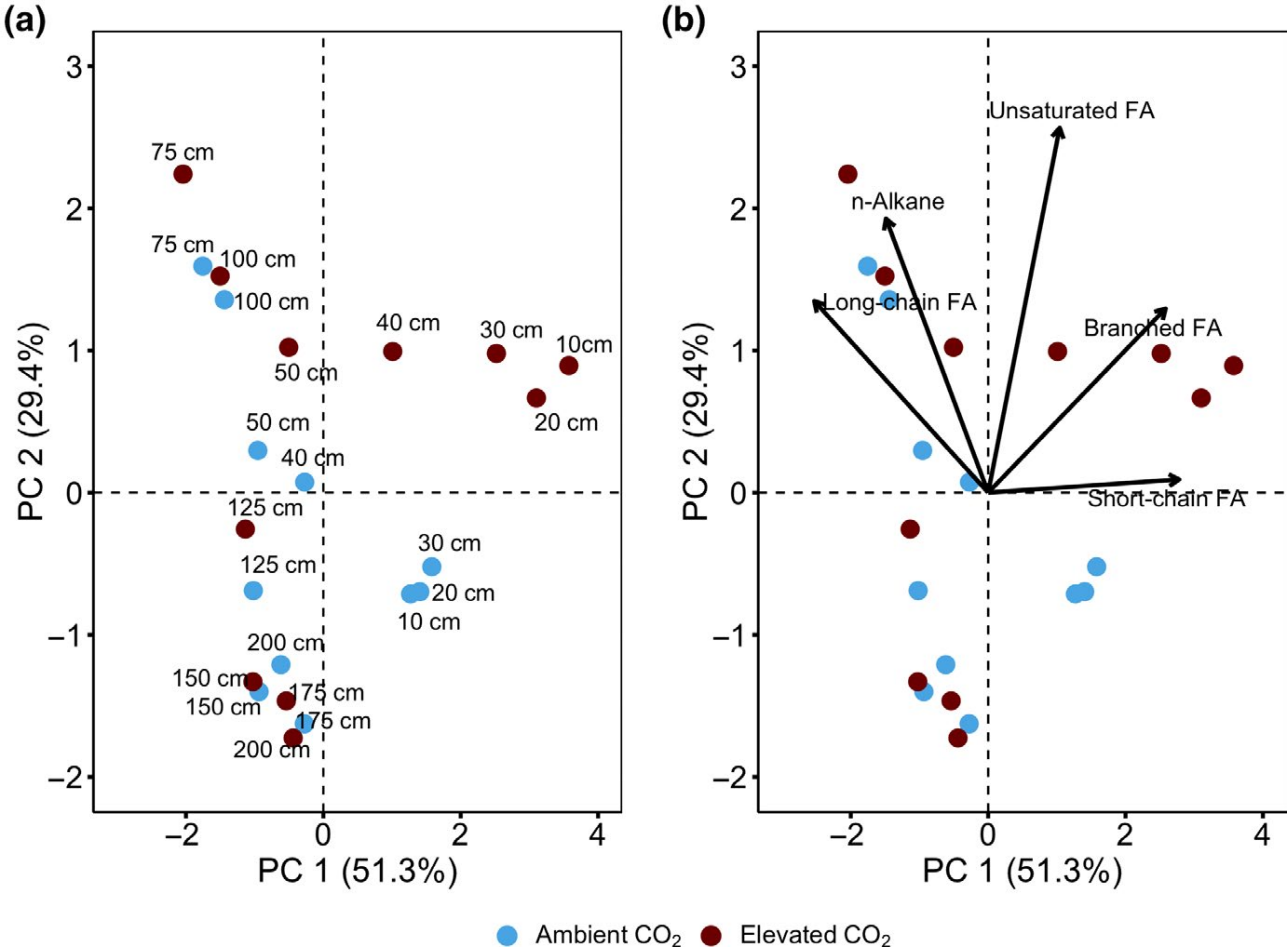
Principal component analysis of biomarkers (a) individual peat depth (from 0 to 200 cm) and (b) biplots of peat depth and biomarkers variables following 4 years of warming and 2 years of elevated atmospheric CO_2_ concentrations. The 0–30 cm depth increment (aerobic acrotelm) clustered according to treatment and was separated from 30 to 75 cm depth (mesotelm) and 75 to 200 cm (anoxic catotelm). The results are expressed as a biplot, where the distance and direction from the axis centre has the same meaning. Numbers in parenthesis represent data variations explained by the first two principal components. Colours represent ambient (blue) or elevated CO_2_ (red) treatment

We also explored changes in OM composition using PCA to understand combined shifts in our biomarker proxies. This analysis confirmed the existence of a distinct composition of biomarkers between the aerobic acrotelm (0–30 cm depth), the mesotelm (30–75 cm depth) and anoxic catotelm (75–200 cm; Figure 6a; Table 2). The PCA also showed a clear separation of aCO_2_ treatments from eCO_2_ treatments in the acrotelm (along PC2; Figure 6a). The first two principal components (PCs) explained 80.7% of the variation of the data (PC 1 = 51.3% and PC 2 = 29.4%). While the variation of the first PC was explained by short‐chain *n*‐FA (*r* = .57, *p* < .0001), branched FA (*r* = .53, *p* < .0001), long‐chain *n*‐FA (*r* = −.52, *p* < .0001) and *n*‐alkane (*r* = −.31, *p* = .022), the variation of the second PC was explained by unsaturated FA (*r* = .69, *p* < .0001), *n*‐alkane (*r* = .52, *p* = .0017) and long‐chain *n*‐FA (*r* = .36, *p* = .041; Figure 6b). These results suggest an accumulation of short‐chain *n*‐FA and branched FA at the expense of long‐chain *n*‐FA and *n*‐alkanes along the peat depth‐profile.

**TABLE 2:** Summary of treatment effects of organic carbon, nitrogen, *n*‐alkane, branched fatty acid (FA), long‐chain and short‐chain *n*‐FA, and carbon preference index (CPI) of alkanes (CPI_ALK_) and fatty acids (CPI_FA_) and average chain length (ACL) of alkanes (ACL_ALK_) and fatty acids (ACL_FA_) and carbon isotope ratios (δ^13^C) of bulk OM, *n*‐alkanes and *n*‐fatty acids (FA)

	Proxy for the following soil process or source	0–30 cm		30–75 cm		75–200 cm	
		Warming	eCO_2_ × warming	Warming	eCO_2_ × warming	Warming	eCO_2_ × warming
Carbon concentration	OM stock/nutrient	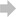	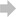	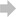	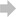	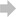	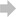
Nitrogen concentration		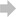	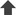	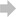	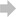	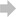	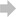
C:N ratio	Degree of decomposition	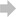	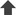	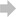	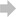	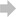	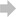
*n*‐Alkane concentration	Plant biomass^a^	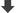	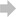	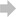	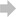	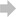	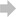
Short‐chain *n*‐FA concentration	Microbial/plant biomass^a^	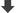	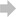	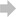	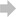	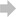	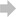
Long‐chain *n*‐FA concentration	Plant biomass^a^	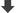	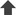	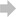	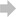	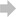	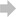
Branched FA concentration	Microbial biomass^a^	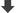	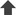	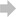	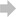	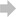	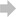
CPI_ALK_	Degree of decomposition	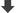	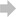	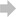	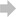	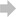	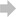
CPI_FA_		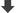	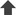	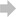	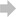	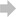	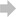
ACL_ALK_	Plant versus microbial biomass	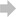	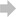	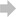	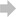	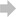	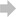
ACL_FA_		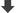	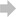	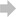	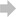	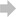	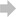
δ^13^C of bulk OM	Pre/post experimental CO_2_	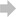	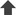	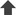	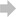	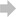	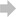
δ^13^C of *n*‐alkanes		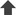	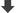	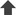	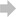	n/a	n/a
δ^13^C of short‐chain FA		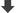	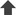	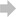	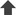	n/a	n/a
δ^13^C of long‐chain FA		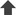	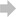	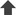	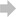	n/a	n/a

Note

Colours represent statistically significant (black) or non‐significant (grey) treatment. Arrows indicate effect, up arrows indicate positive treatment effects, sideways arrows indicate no effect, and down arrows indicate negative treatment effects, n/a indicates that treatment effects cannot be determined.

^a^ These proxies can reveal trends, but they do not originate exclusively from either plant or microbial biomass.

## 4 DISCUSSION

Experimental warming and elevated atmospheric CO_2_ concentration strongly altered OM quality and quantity. Using a combination of isotopic and biomarker analyses on peat C characteristics, we found that warming and elevated atmospheric CO_2_ concentration had divergent ecosystem effects. Warming‐only treatments increased OM degradation while the interaction between warming and elevated CO_2_ concentration increased OM incorporation of newly produced plant biomass inputs. The effects of increased plant inputs may progressively shift boreal peatland OM composition and ultimately, C sequestration potential under rising temperature and atmospheric CO_2_ concentration.

### 4.1 Depth‐specific transformation of peat organic matter

Our results provide evidence that warming and elevated CO_2_ concentrations increased the incorporation of plant‐derived OM to the aerobic acrotelm (0–30 cm depth; Figures 2, 4 and 6; Table 2). This effect sharply attenuated with depth (Figures S1–S3; Table 2), mainly due to the anoxic conditions in the water‐saturated catotelm (75–200 cm depth), which limit OM decomposition (Dorrepaal et al., 2009; Freeman et al., 2001; Moore and Basiliko, 2006). One simple reason for depth‐specific trends could be that since the catotelm underwent decomposition when it was part of the aerobic acrotelm (a thousand years ago; 1580–9200 bp; Hobbie et al., 2016), its chemical composition may be less susceptible to the changes observed in the acrotelm (Frolking et al., 2010).

We further observed accelerated OM decomposition and peat C cycling in the acrotelm (Figures 2, 4 and 6; Table 2). At the SPRUCE experimental site, warming resulted in a limited water level drawn down to about 30 cm depth below the hollow in the warmer plots during summer dry periods (Hanson et al., 2020), even though ample precipitation inputs throughout the early years of the SPRUCE study limited water table reductions during the tenure of this study. Therefore it is plausible that redox oscillations facilitated by changes in the water table levels (Hanson et al., 2020; Tfaily et al., 2018), may be responsible for the observed OM transformation. These findings provide a direct measure of OM degradation in ombrotrophic peatlands and support previous studies that hypothesize a change in C cycling due to climate change in peatlands (Bridgham et al., 2008; Chen et al., 2008; Wilson et al., 2016). Moreover, our depth‐specific results (Figure 6) emphasize the critical interactive impact of temperature and water level in regulating ecosystem carbon accumulation and greenhouse gas exchange in bog and fen peatlands. As of now, 4 years into the treatment, the catotelm appears to be stable under the current chemical and hydrological conditions, although this may change in the future (Hopple et al., 2020). Additionally, it might be the mesotelm, which is exposed to seasonal variations in water table and, thus, oxidizing and reducing conditions, that may show a long‐term change to global warming. Although not available for this study, the dramatic drought conditions of 2021 open up the possibility for further evaluation of this hypothesis.

### 4.2 Elevated CO_2_ increased plant inputs but not peat organic matter in unwarmed treatment

Contrary to our hypothesis, the unwarmed elevated CO_2_ treatment did not increase peat C concentrations after 2 years (Figure 1a). Instead, we observed loss of peat nitrogen concentrations (+0°C warming; Figure 1b) in the acrotelm, implying higher nitrogen demand likely due to enhanced vegetation productivity from CO_2_ fertilization (McPartland et al., 2019). Such changes would be expected to enhance the incorporation of plant‐derived OM into peat C (Walker et al., 2020), but so far, we do not observe this in the peat C stocks. However, a potential faster cycling of new carbon inputs (Van Groenigen et al., 2014; Walker et al., 2020), supported by the observed rapid incorporation of new labelled C in our study (Figures 4 and 5), cannot be ruled out and is likely limiting the potential for C storage. Not only did the elevated CO_2_ treatment deplete ^13^C abundances relative to ambient CO_2_ treatment, as per our expectation (Figure 4; Figure S5), but biomarker proxies under elevated CO_2_ treatment also had similar δ^13^C values to those of plant leaves (Table 1). This finding suggests a rapid incorporation of plant inputs into peat C in the acrotelm (Diefendorf et al., 2015; Liu & An, 2020; Naafs et al., 2019). Thus, our observation that after 2 years of unwarmed‐elevated CO_2_ treatment, plant‐derived OM inputs into peat C increased without increasing peat C stocks, suggests that peatland C cycling may be accelerating, although this remains to be directly tested. However, our results corroborates previous studies positing that increased plant biomass inputs related to greater CO_2_ concentrations can cause an enhanced supply of easily metabolized substrates, in turn stimulating the decomposition of longer‐time preserved OM and limiting belowground C storage (e.g., Van Groenigen et al., 2014).

### 4.3 Accelerated organic matter decomposition in a warmer acrotelm

We hypothesized that warming would accelerate peat decomposition through enhanced microbial activity (Gill et al., 2017; Hanson et al., 2020), especially at the warmest end of the SPRUCE temperature gradient. Indeed, we observed stronger decomposition in the active acrotelm of the warming‐only treatments (ambient CO_2_), as reflected by the biomarker‐based proxies for OM degradation (Figures 2 and 3). Accelerated decomposition was suggested in previous SPRUCE observations of warming‐ and drying‐induced increases in CO_2_ and CH_4_ emissions (Wilson et al., 2016). Building on this, our study provides direct evidence that degradation accelerated with warming and highlights that the majority of these changes take place in the acrotelm (Figures 2, 3, 4; Table 2).

Our interpretation of warming induced increase in OM degradation is based on a variety of proxies. We observed that OM decomposed faster, irrespective of their chemical structure (Figure 2). Soil constituents such as FAs have traditionally been considered to represent easier decomposable components of OM (Pisani et al., 2015), than, for example, *n*‐alkanes (Hedges et al., 1988; Rumpel et al., 2002). However, not only did the biomarker proxies become enriched in ^13^C with increasing temperature, as per our expectation (Figure 4; Figure S5), also their concentrations decreased rapidly (Figure 2), implying accelerated decomposition of all measured biomarkers (Figure 2). Thus, our observations confirm that there is no simple relationship between OM molecular structure, decomposition and warming (Lehmann & Kleber, 2015). These results combined, show that C components traditionally believed to cycle slowly, on a multi‐decadal time scale (e.g. Li et al., 2019; Pisani et al., 2015), can respond rapidly, over a few vegetation periods to decades (Schmidt et al., 2011). The warming lowered the water table (discussed in Section 4.1), and the resulting oxygenation probably enhanced incorporation of different soil constituents (such as protein and sugars; e.g. Dieleman et al., 2016; Pold et al., 2017) via plant litter and root exudates, accelerating the decomposition of fast cycling C and dilution of slowly cycling C. We cannot rule out that the artificial step change caused by the start of the temperature gradient also contributed to the observed strong changes in peat OM. The finding that presumably slowly cycling peat C responded so rapidly to warming is novel, and highlights that peat C, can be vulnerable to rising temperatures.

Taken together, the above findings indicate that direct acceleration of microbial decomposition, combined with the lowering of the water table due to increased temperature (Schlesinger & Bernhardt, 2020) will increase oxygen availability and accelerate microbial decomposition of peat C in the previously water saturated zones (Schlesinger & Bernhardt, 2020; Yu, 2012) despite increased C input (Hanson et al., 2020).

### 4.4 The counteractive effect of warming and elevated CO_2_ on peat organic matter

We initially hypothesized that warming combined with an elevated atmospheric CO_2_ concentration would not alter peat C composition given the limited evidence of CO_2_ effect so far at the nutrient‐limited SPRUCE experiment (e.g., Hanson et al., 2020; Hopple et al., 2020; Wilson et al., 2016). Yet, our results provide evidence that the interaction of warmer temperatures and greater CO_2_ concentrations leads to increased plant C inputs within the acrotelm (Figure 2), more than warming treatments by themselves (i.e. carbon loss) or elevated CO_2_ treatments without warming (no detectable response; Figure 2a,b). Not only did we observe increased concentrations of the biomarker proxies under combined treatment of warming and CO_2_ enrichment (Figure 2b,c) but also the biomarker proxies become enriched in ^13^C with increasing temperature (Figure 4b), implying that the new C inputs were more strongly offsetting carbon losses that occurred under warming‐only treatment. Our results thus emphasize the counteractive effects of warming and elevated atmospheric CO_2_ concentration on composition and decomposition of peat C and shows that different, competing mechanisms can govern peatland C dynamics. Decomposition processes dominate under rising temperature, whereas incorporation of plant inputs into peat C increase when rising temperatures are combined with elevated atmospheric CO_2_ concentrations (Ainsworth & Long, 2021; Leakey et al., 2009; Song et al., 2019; Walker et al., 2020).

It is important to recognize that changes in temperature and atmospheric CO_2_ concentration may stimulate productivity of vascular plants but reduce peat‐moss formation (Bragazza et al., 2013; Norby et al., 2019). As the source of C input changes, so does the decomposability of the input given that mosses have much lower decomposability relative to vascular species (Moore et al., 2007). The ^13^C labelling and tracking of elevated CO_2_‐derived carbon as a measure for C cycling (Walker et al., 2020) revealed that warming and CO_2_ enrichment stimulated rapid incorporation and accumulation of new C in the short term (Figure 5), likely from the combination of decreased bryophyte production (Norby et al., 2019) and increased vascular plant biomass (Malhotra et al., 2020; McPartland et al., 2019). Indeed, the biomarker‐based proxies for OM degradation did not differ between elevated CO_2_ treatments (Figure 3b; Figure S4) and were similar to those observed in the plant biomass (Table 1), indicative for fresh OM input. The source could be roots because root litter production and incorporation have been shown to significantly increase under elevated CO_2_ concentration especially with low nutrient availability (Ainsworth & Long, 2021; Song et al., 2019). Furthermore, biomarker‐based proxies for microbe‐derived OM had higher fractions of new C than plant‐derived OM (Figure 5; Figure S6), likely due to the rapid incorporation of root exudates and microbe‐derived OM with a faster cycling (Kramer & Gleixner, 2006; Wiesenberg et al., 2008). Other ecosystem‐scale experiments showed inconsistent responses to elevated CO_2_, either an increase (Iversen et al., 2012) or no change (Girardin et al., 2016; Hoosbeek et al., 2001) in carbon input belowground.

From our results, we conclude that in peatland ecosystems different processes occur with warming, elevated atmospheric CO_2_ concentration and their interaction. The net increase in new peat C in the active acrotelm with warming and elevated CO_2_ treatment might reflect a transient adjustment period to the instantaneous applications of the SPRUCE warming treatments where microbial activity and decomposition rates have not yet reached a new equilibrium. Although faster C cycling within extant peat C pools themselves cannot be ruled out and are likely to have occurred, they appear to have been offset by more plant inputs and decreased susceptibility to decomposition, without necessarily altering overall C storage under the warming and elevated CO_2_ treatments (Figure 1a).

### 4.5 Implications: Rising temperatures and atmospheric CO_2_ could cause net C loss

Boreal ecosystems are warming faster than the global mean (Bekryaev et al., 2010), and they are projected to warm by 4–6°C by 2100, under RCP 8.5 scenario (Soong et al., 2020). Here, we demonstrate that the peat C pool is sensitive to future climate change. Specifically, the complementary biomarker‐based proxies revealed divergent responses to rising temperatures and atmospheric CO_2_ levels on different peat C pools previously not predicted by ecosystem experiments. Decomposition increases dominated the warming response, whereas plant and microbial inputs (via microbial utilization of new photosynthates) increased when warming and elevated atmospheric CO_2_ concentration were combined. These responses were more pronounced in the surface aerobic acrotelm than in the catotelm, highlighting the sensitivity of the aerobic layer OM to changing environmental conditions.

Although several peatland climate change experiments exist, and biomarkers are an established tool to understand ecosystem processes (Jansen & Wiesenberg, 2017), investigations on peat biomarker responses are scarce. Moreover, our technique applied in SPRUCE’s unique whole‐ecosystem warming setting, allowed us to understand how different components of peat C (bulk C vs. individual OM components) responded differently to warming and elevated atmospheric CO_2_ concentrations. Although our results are consistent with the prevailing view that rising temperatures and atmospheric CO_2_ levels will likely enhance the terrestrial feedback on climate in boreal peatlands (Bragazza et al., 2013; Hopple et al., 2020; Wilson et al., 2016), it remains unclear whether a system shift characterized by more rapid carbon cycling will be created by increased vascular plant productivity and loss of the ecosystem‐engineer mosses (Malhotra et al., 2020; Norby et al., 2019), and whether the observations from our experimental site are representative for all northern peatland types. It is important, therefore, to complement our results with long‐term, time‐resolved analyses and to apply our results into revised parameterizations for OM dynamics in SPRUCE model frameworks (Golaz et al., 2019). Future efforts like these will allow us to test if the findings are generalizable and to improve confidence in future projections of OM dynamics. Lastly, our observed counteractive effects of the impacts of elevated atmospheric CO_2_ conditions on rising temperatures call for caution when extending predictions of warming impacts based on observations under ambient CO_2_ conditions and demonstrates the need of multifactorial experiments to inform future models.

In summary, rising temperatures and atmospheric CO_2_ levels are expected to directly affect C cycling in peatlands by altering OM inputs, quality and quantity, thus influencing the decomposition parameters that dictate peatland carbon storage (Frolking et al., 2010). Peatlands build carbon stocks over centuries, but rising temperatures and atmospheric CO_2_ concentrations at SPRUCE changed the equilibrium within only 2 years, highlighting the vulnerability of these C rich ecosystems to global change.

## CONFLICT OF INTEREST

The authors declare no competing interests.

## AUTHOR CONTRIBUTIONS

Paul J. Hanson designed and maintained the warming field experiment. Michael W. I. Schmidt conceived the DEEP C project. All co‐authors participated in the field campaign and data interpretation and contributed actively to the manuscript written by Nicholas O. E. Ofiti. Nicholas O. E. Ofiti carried out biogeochemical analyses.

## Supporting information

Supplementary Material

## Data Availability

The data used in this study will be available in the online project archive at https://mnspruce.ornl.gov and the long‐term storage in the U.S. Department of Energy's Environmental Systems Science Data Infrastructure for a Virtual Ecosystem (ESS‐DIVE; https://ess‐dive.lbl.gov).
