# Supplementary material for: Warming and elevated CO2 promote rapid incorporation and degradation of plant‐derived organic matter in an ombrotrophic peatland
Source: Glob Chang Biol. 2021 Nov 8;28(3):883–98. doi: 10.1111/gcb.15955 (PMC9299048; doi:10.1111/gcb.15955)

Warming and elevated CO_2 promote rapid incorporation and accelerated degradation of plant-derived organic matter in an ombrotrophic peatland

Nicholas O.E. Ofiti[1*], Emily F. Solly[2], Paul J. Hanson[3], Avni Malhotra[1], Guido LB Wiesenberg[1], and Michael W.I. Schmidt[1]

[1]Department of Geography, University of Zurich, Zurich, Switzerland
[2]Group for Sustainable Agroecosystems, Department of Environmental Systems Science, ETH Zurich, Zurich, Switzerland
[3]Environmental Sciences Division and Climate Change Science Institute, Oak Ridge National Laboratory, Oak Ridge, TN, USA

***Corresponding author:** Email: nicholas.ofiti@geo.uzh.ch

Supplemental Materials

Table. s1. Environmental conditions in the experimental enclosures.

Plot #	CO_2 treatment	Nominal warming treatment	CO_2 (ppm)	Average soil temperature at 30 cm (°C)
6	Ambient	+0	423	4.0
19	Elevated	+0	708	5.5
20	Ambient	+2.25	415	6.9
11	Elevated	+2.25	698	7.1
13	Ambient	+4.5	415	8.1
4	Elevated	+4.5	723	8.8
8	Ambient	+6.75	416	9.6
16	Elevated	+6.75	743	10.4
17	Ambient	+9	429	11.4
10	Elevated	+9	736	12.1

Note: Soil temperature was measured at -0.3 m below the hollows averaged over the period 2016 to 2018.

Fig. s1. Peat organic matter responses to warming and elevated CO_2, a) organic carbon, and b) nitrogen concentrations, c) bulk $\delta^{13}C$ and d) C:N ratio, following 4 years of warming and 2 years of CO_2 addition. Colours represent ambient (grey) or elevated CO_2 (black) treatment (+500 ppm above ambient). Symbols represent different sampling depth. Lines indicate significant treatment effects (regression smooth curves) $p < 0.05$. Linear regression with 95% confidence intervals is shown in grey. The absence of a line and/or confidence intervals indicates no significant trend.

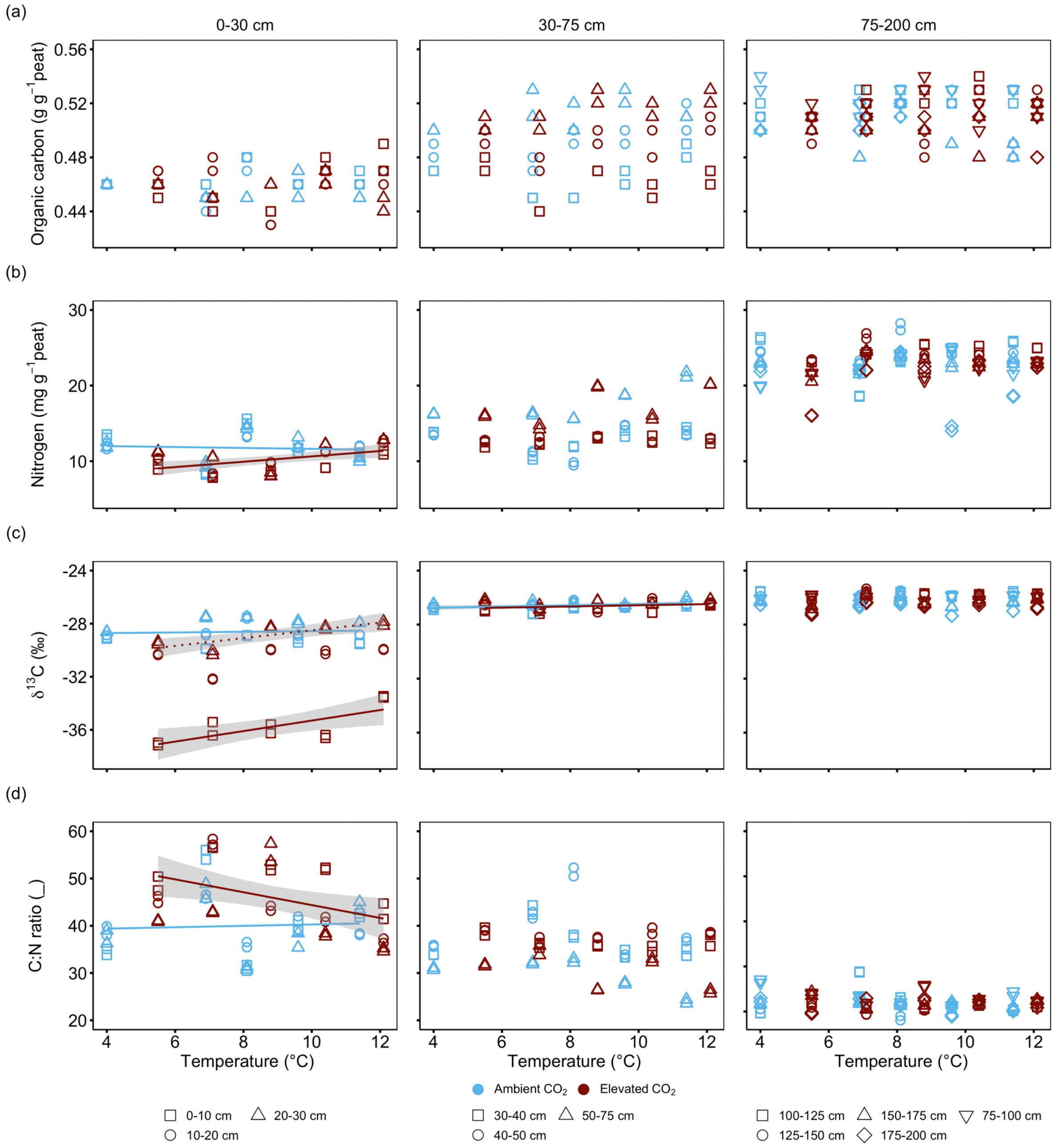

Fig. s2. Relative abundance of a) *n*-Alkanes (C_{23-35}), and b) Long-chain *n*-fatty acids (C_{20-32}) concentrations following 4 years of warming and 2 years of CO_2 addition. Colours represent ambient (grey) or elevated CO_2 (black) treatment (+500 ppm above ambient). Symbols represent different sampling depth. Lines indicate significant treatment effects (regression smooth curves) $p < 0.05$. Linear regression with 95% confidence intervals is shown in grey. The absence of a line and/or confidence intervals indicates no significant trend.

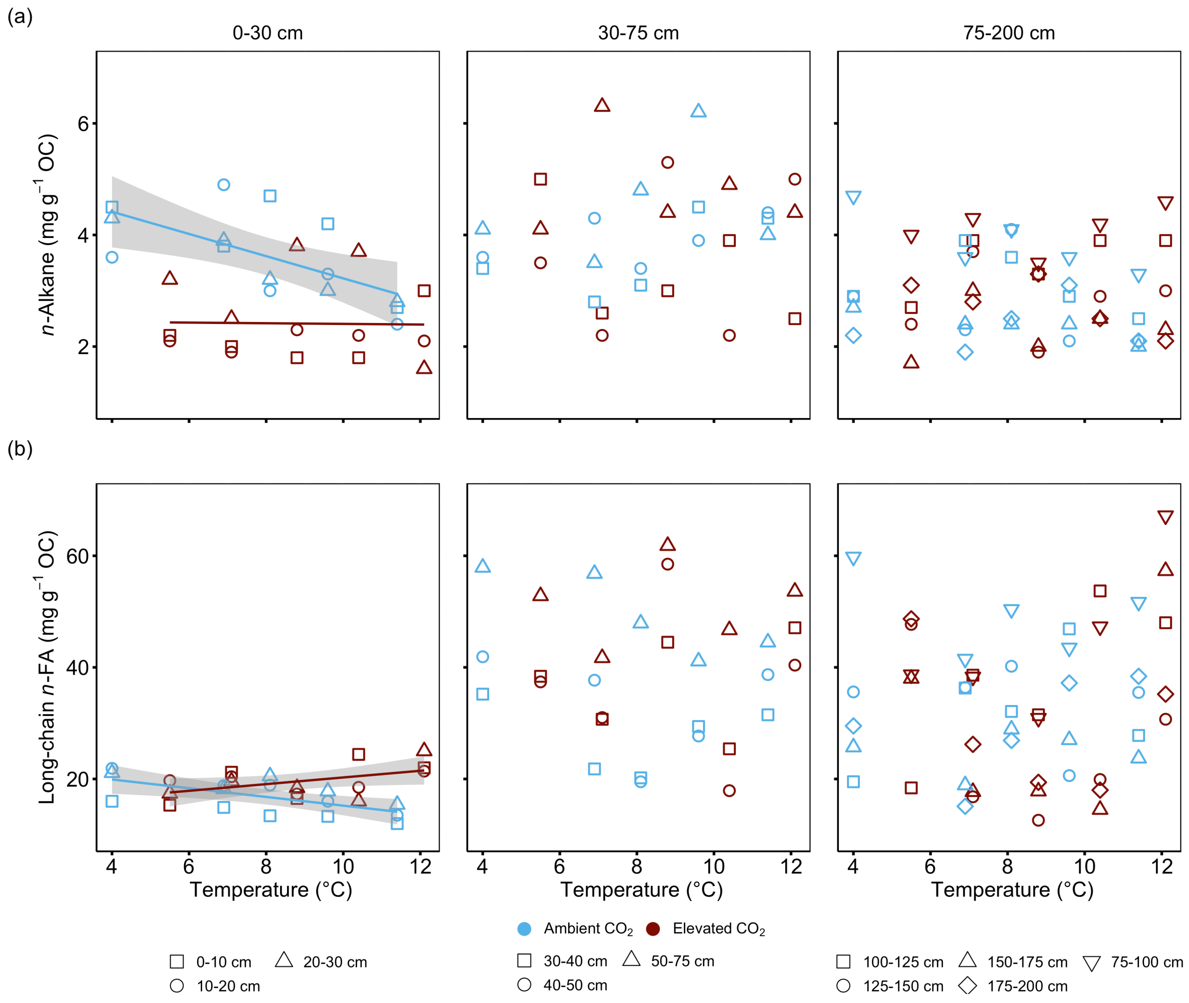

Fig. s3. Relative abundance of a) Short-chain fatty acids (C_{12-18}), b) branched fatty acids (*iso*-C_{15}, *anteiso*-C_{15}, *iso*-C_{16}, *iso*-C_{17}, *anteiso*-C_{17}, *cis9,10*-cy-C_{16}, *cis9,10*-cy-C_{18} and $C_{18:2\omega6}$), and c) unsaturated fatty acids ($C_{16:1,\ 18:2,\ 18:1}$) concentrations following 4 years of warming and 2 years of CO_2 addition. Colours represent ambient (grey) or elevated CO_2 (black) treatment (+500 ppm above ambient). Symbols represent different sampling depth. Lines indicate significant treatment effects (regression smooth curves) $p < 0.05$. Linear regression with 95% confidence intervals is shown in grey. The absence of a line and/or confidence intervals indicates no significant trend.

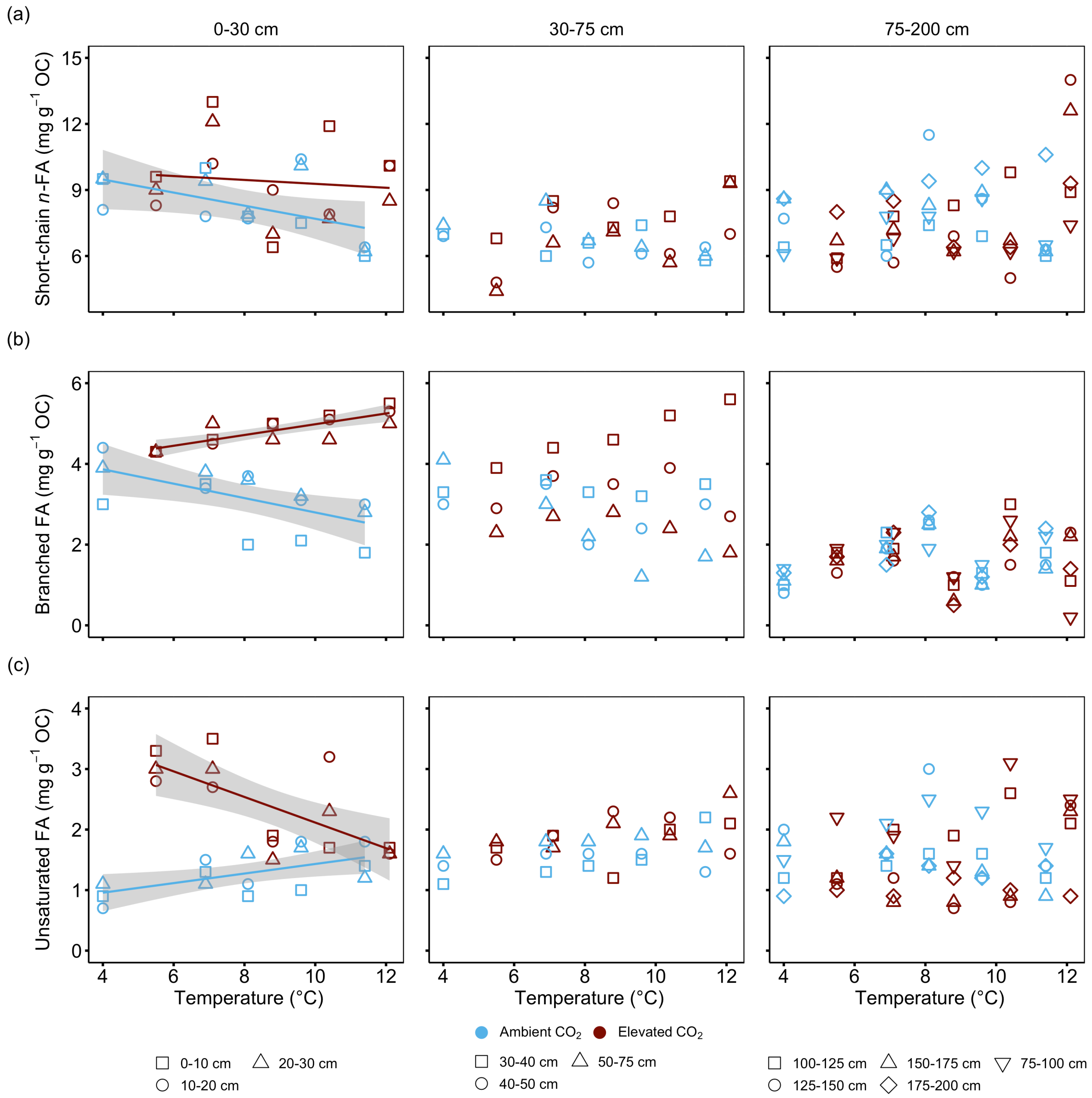

Fig. s4. Decomposition proxies of average chain length (ACL) of a) *n*-Alkanes (ACL_{ALK}) and b) fatty acids (ACL_{FA}) and carbon preference index (CPI) of c) *n*-Alkanes (CPI_{ALK}) and d) fatty acids (CPI_{FA}) following 4 years of warming and 2 years of CO_2 addition. Colours represent ambient (grey) or elevated CO_2 (black) treatment (+500 ppm above ambient). Symbols represent different sampling depth. Lines indicate significant treatment effects (regression smooth curves) $p < 0.05$. Linear regression with 95% confidence intervals is shown in grey. The absence of a line and/or confidence intervals indicates no significant trend.

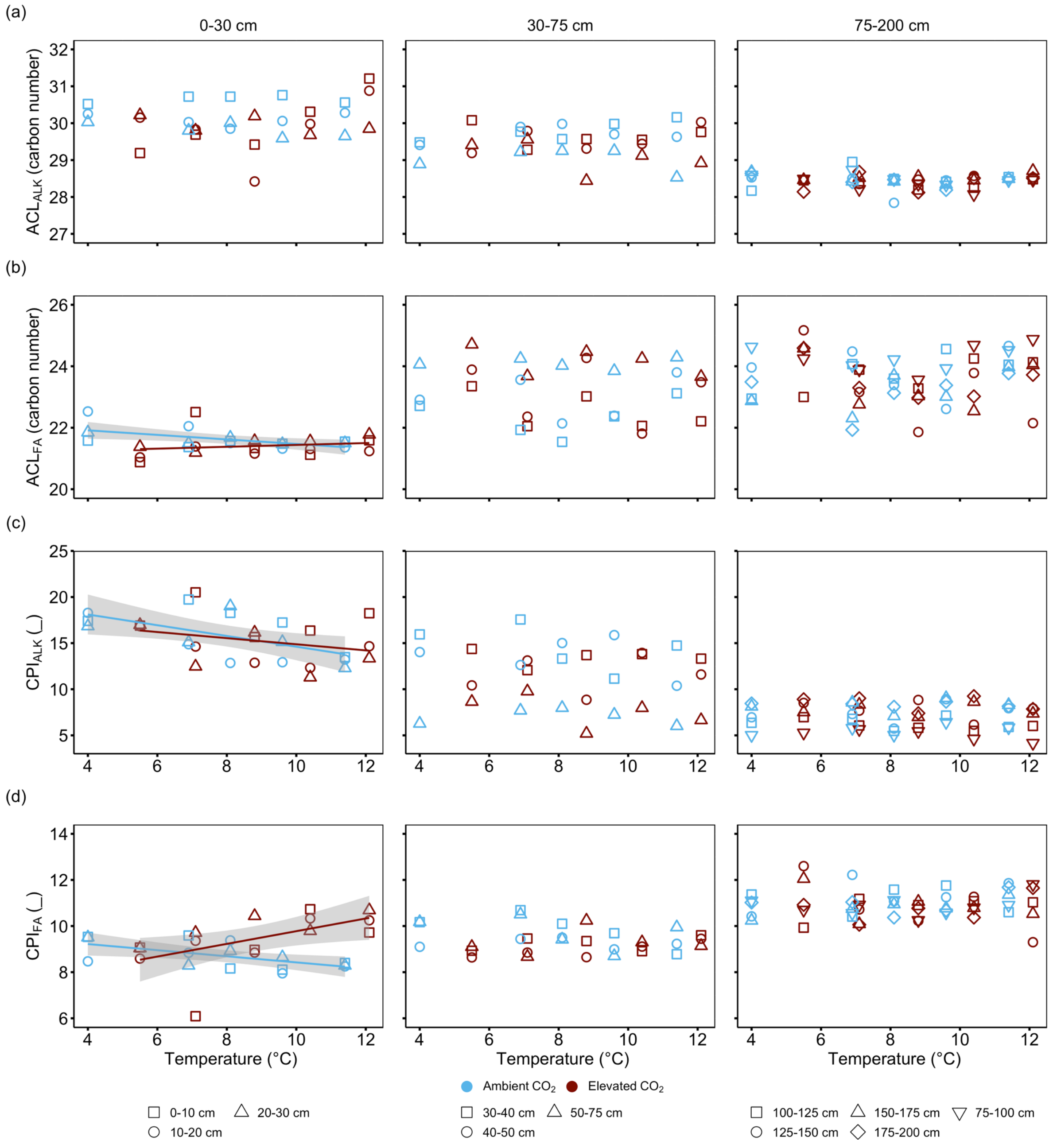

Fig. s5. Compound-specific δ13C values ratios of the most abundant a) *n*-alkanes $C_{23–33}$) b) short-chain *n*-fatty acids ($C_{14–18}$) and c) long-chain *n*-fatty acids ($C_{20–32}$) following 4 years of warming and 2 years of CO_2 addition. Colours represent ambient (grey) or elevated CO_2 (black) treatment (+500 ppm above ambient). Symbols represent different sampling depth. Lines indicate significant treatment effects (regression smooth curves) $p < 0.05$. Linear regression with 95% confidence intervals is shown in grey. The absence of a line and/or confidence intervals indicates no significant trend.

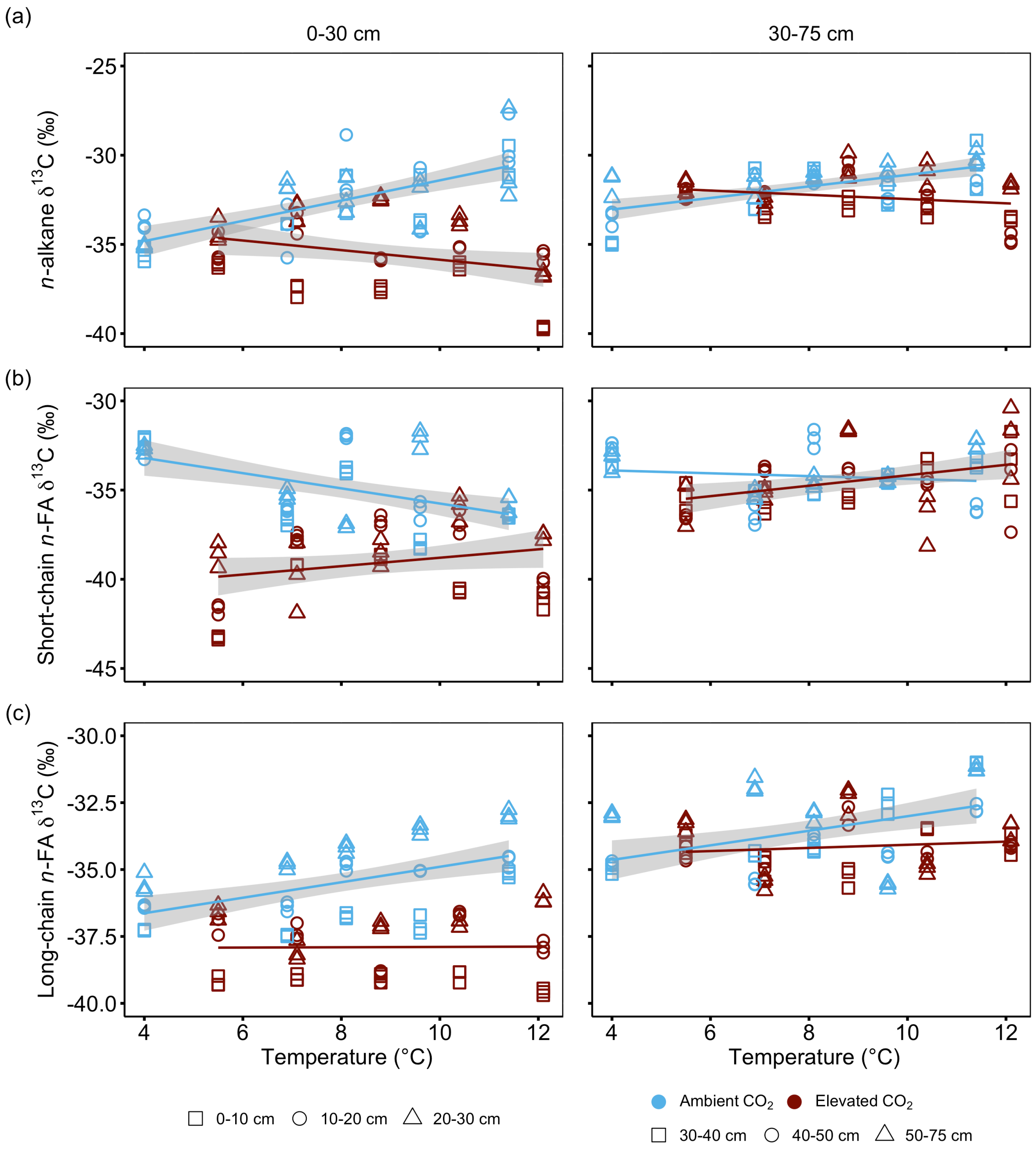

Fig. s6. Fractions of new (experiment-derived) carbon in a) *n*-Alkane C_{23-33}), b) short-chain *n*-fatty acids (C_{14-18}) and c) long-chain *n*-fatty acids (C_{20-32}) after 4 years of warming and 2 years of elevated atmospheric CO_2 concentrations. Symbols represent different sampling depth. Lines indicate significant treatment effects (regression smooth curves) $p < 0.05$. Linear regression with 95% confidence intervals is shown in grey. The absence of a line and/or confidence intervals indicates no significant trend.

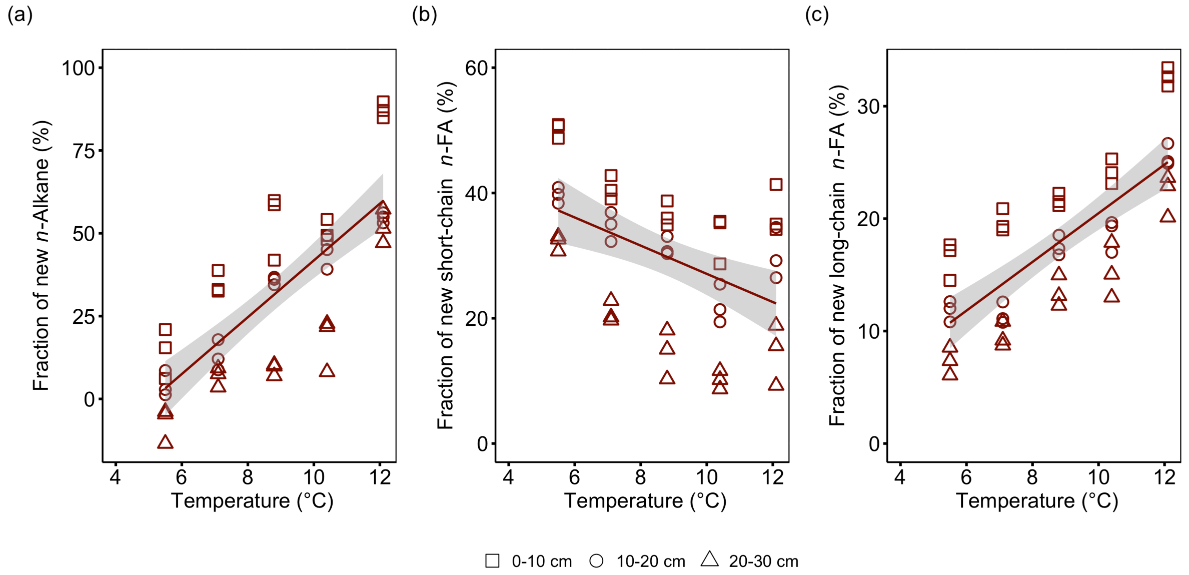

Supplement: Supplementary file 1 — Supplementary Material [file GCB-28-883-s001.pdf]
